# Hybrid vigor and outbreeding depression after 100 years of replicate introduction of Caucasian beech to European forests

**DOI:** 10.1038/s41437-026-00860-7

**Published:** 2026-08-10

**Authors:** Camilla Stefanini, Christoph Sperisen, Igor Chybicki, Frédéric Guillaume, Mirjam Kurz, Katalin Csilléry

**Affiliations:** 1 Evolutionary Genetics Group, Biodiversity and Conservation Biology, Swiss Federal Research Institute WSL, Birmensdorf, Switzerland; 2 Forest Health and Biotic Interactions, Swiss Federal Research Institute WSL, Birmensdorf, Switzerland; 3 Department of Genetics, Kazimierz Wielki University, Bydgoszcz, Poland; 4 Department of Organismal and Evolutionary Biology, University of Helsinki, Helsinki, Finland; 5 Institute of Natural Resource Sciences, Zurich University of Applied Sciences ZHAW, Wädenswil, Switzerland

**Keywords:** Ecological genetics, Plant hybridization

## Abstract

Hybridization between closely related forest tree species is a major concern for assisted migration decisions. Historical introductions, such as the repeated introduction of Caucasian beech (*Fagus hohenackeriana*) into European beech (*Fagus sylvatica*) forests over 100 years ago, provide insight into potential outcomes. In two such forest stands in Central Europe, we exhaustively genotyped adults (*N* = 216 and 226) and surrounding seedlings and juveniles (*N* = 639 and 423). We found that the two species interbred successfully, with interspecific F1 hybrids comprising 10.4% and 34.2% of the seedlings in the two stands, respectively. However, their proportion decreased in later age classes, and advanced-generation hybrids were rare (<4%), despite up to three possible generations of introgression. Parentage analyses revealed a tendency toward assortative mating, likely driven by the, on average, 2.3-day earlier spring bud break of Caucasian beech, based on five years of observations. In agreement with this finding, F1 seedlings grew near adult Caucasian beeches, which acted as seed donors. To assess whether these patterns could arise from demographic processes alone or reflect outbreeding depression, we used spatially explicit individual-based simulations with mating and demographic parameters specific to the study system and sites, including selection against F1s. All simulated scenarios underestimated F1 frequencies and overestimated advanced-generation hybrid frequencies, suggesting that life-stage-specific selection may alter hybrid persistence. We conclude that Caucasian beech introductions do not pose a risk of invasiveness, and that mild seedling-stage-specific heterosis effect overridden by outbreeding depression at the adult stage is the most parsimonious explanation for the observed patterns. Our simulation pipeline can be used for other species and contexts to evaluate potential outcomes of assisted gene flow.

## Introduction

Hybridization has long been recognized as a major mechanism driving both speciation and adaptation to novel environments (Arnold 2006; Rieseberg 1997; Seehausen 2004). More than 25% of plant species hybridize with at least one other species (Mallet 2005), and widespread hybridization is also increasingly reported in animals (Abbott et al. 2013). Although genomic studies have illuminated the long-term evolutionary consequences of hybridization, its short-term fitness effects, including heterosis and outbreeding depression, remain comparatively less well documented in natural systems, partly because they require controlled crosses or multi-generation observations that are challenging and time-consuming to conduct. This gap is particularly relevant in the context of Assisted Gene Flow (AGF) and genetic rescue, which are increasingly proposed as tools to maintain genetic diversity and promote adaptation in forest ecosystems (Aitken and Bemmels 2016). The effectiveness of these interventions depends on our understanding of the short- and medium-term outcomes of hybridization (Aitken and Whitlock 2013; Grummer et al. 2022). In plants, genetic rescue can restore fitness in small, inbred populations through increased cross-compatibility and heterosis (Willi and Fischer 2005), but its benefits may be more limited where local adaptation constrains the performance of introduced genotypes (Rousselle et al. 2011).

In the short term or in later generations, hybridization between genetically divergent individuals can lead to outbreeding depression, the reduction in offspring fitness relative to the parental populations (Edmands 2007; Frankham et al. 2011a). Reduced fitness may already occur in the F1 generation, or emerge in subsequent generations as recombination disrupts co-adapted gene complexes, a process often referred to as hybrid breakdown (Aitken and Whitlock 2013). Such effects may arise through incompatible allelic interactions, including Dobzhansky-Muller incompatibilities (Dobzhansky 1937). While outbreeding depression has been documented across multiple generations in short-lived species (Dolgin et al. 2007; Fenster and Galloway 2000), it remains poorly characterized in long-lived species such as forest trees, where extended generation times make it challenging to follow hybrid fitness across life stages. Yet the fitness consequences may become apparent only later in life, such as during adult growth, survival, or reproduction. For example, reciprocal crosses between local and non-local populations of *Abies sachalinensis* showed reduced growth and possibly lower reproductive success in mature individuals (Goto et al. 2011). Despite these potential delayed effects, most empirical and simulation studies have focused primarily on F1 hybrids rather than on the fate of hybrid offspring across generations or through ontogeny (Aitken and Whitlock 2013); see, however, Irwin and Schluter (2022). This gap highlights the need for studies of long-lived trees that track hybridization outcomes across the full life cycle.

Hybridization can also produce the opposite outcome: increased fitness in early hybrid generations (heterosis). Heterosis has been extensively exploited in agriculture and forestry, particularly in crops and fast-growing trees such as poplar and eucalyptus (Clifton-Brown et al. 2019; Fu et al. 2014; Labroo et al. 2021; Li et al. 2024). In forest trees, heterosis has also been reported in hybrids of pine (Dungey 2001), larch (Marchal et al. 2017), and other conifers (MacLachlan et al. 2018). For example, crosses between Japanese chestnut (*Castanea crenata*) and Chinese chestnut (*Castanea mollissima*) successfully combine disease resistance with high productivity (Craddock and Perkins 2020). Evidence from natural populations also suggests that heterosis may enhance adaptive potential and resilience to environmental stress, as observed in poplar, oak, and pine (Hord et al. 2025; Leroy et al. 2020; Menon et al. 2021). Yet, in long-lived species, empirical evidence remains limited, as fitness advantages may emerge only at specific life stages or under particular environmental conditions. Early work in the genus *Fagus* suggested that seedlings from artificial crosses between *F. sylvatica* and *F. orientalis* exhibited enhanced growth consistent with heterosis (Nielsen and Muckadell 1954). Overall, the magnitude and persistence of heterosis depend on the traits measured and the environmental context, and its importance in natural tree populations remains poorly understood.

Understanding the short- and medium-term consequences of hybridization is therefore relevant for evaluating proposals of AGF in forest trees (Aitken and Bemmels 2016; Hällfors and Dalrymple 2022). Because AGF intentionally introduces genotypes from other populations or regions, it may generate hybridization between genetically divergent lineages, potentially producing both heterosis and outbreeding depression. Although support for implementing AGF is growing (Chakraborty et al. 2024), its long-term genetic and ecological consequences remain underexplored in natural tree populations, largely because of their long life cycles and complex demographic structures. Historical introductions of foreign tree species offer valuable systems to investigate these processes. For instance, the introduction of Caucasian beech (*Fagus hohenackeriana*) into European beech (*Fagus sylvatica*) forests a century ago has resulted in viable hybrid offspring (Budde et al. 2023; Kurz et al. 2023). These systems provide an opportunity to address key evolutionary genetic questions relevant to AGF, including whether hybridization confers fitness advantages that could promote the spread of introduced genotypes, and whether reduced hybrid fitness in later generations (outbreeding depression) may counterbalance any initial gains.

Here, we investigate the evolutionary consequences of hybridization between European beech and introduced Caucasian beech using two historical planting sites for which detailed background information is available (Kurz et al. 2023). Using genetic data across multiple life stages (seedlings, saplings, juveniles and adults), we test three expectations relevant to the evolutionary outcomes of AGF. First, if the introduced species or its hybrids have higher fitness, we expect higher reproductive success and an increasing representation of their genotypes across generations. Second, if mating is unbiased, hybridization rates should correspond to expectations under random mating, whereas deviations from these expectations may indicate assortative or disassortative mating that could influence introgression (Dodet and Collet 2012). Thus, we also examined spring phenology of the two species to assess whether differences in flowering timing could influence mating patterns and contribute to asymmetric or disassortative hybridization. Third, if hybrid fitness is reduced, hybrid genotypes should decline across life stages relative to neutral expectations. To evaluate this last prediction, we compare the observed distribution of hybrid classes with expectations generated by individual-based simulations informed by the historical planting scenario and system-specific mating parameters.

## Materials and methods

### Study system and sampling

European beech (*Fagus sylvatica L*.) is a foundation species of European temperate forests. The closely related Caucasian beech (*F. hohenackeriana* Palibin; (Denk et al. 2024)) was introduced to several stands in the Rhine Valley (Kurz et al. 2023). We focused on two such mixed stands described in Kurz et al. (2023), located near the France-Germany-Switzerland border: Wäldi (Thurgau, Switzerland, $4{7}^{\circ }37{\prime} 44.6{\prime\prime} N$, ${9}^{\circ }06{\prime} 17.9{\prime\prime} E$, 3.3 ha) and Allenwiller (Vosges Mountains, France, $4{8}^{\circ }38{\prime} 33.1{\prime\prime} N$, ${7}^{\circ }21{\prime} 50.5{\prime\prime} E$ 6.3 ha).

In Wäldi, Caucasian beech trees were introduced around 1921 following the coppice-with-standards management system of an oak stand. Beech trees were subsequently favored during the final coppicing rotation in 1950, allowing them to reach the upper canopy (Ulrich, 2017). Today, six of the original Caucasian beech trees remain, clustered in the northwestern part of the site within an area of approximately 90 × 50 m, surrounded by European beech. In Allenwiller, seedlings of both species, of unknown number and origin, were planted between 1910 and 1912 beneath sessile oaks managed using the same silvicultural system (Klein, 1981). At present, most adult Caucasian beech trees occur in a triangular area of approximately 0.5 ha. Both stands have experienced regular at thinning approximately 10-year intervals. The most recent thinning occurred in 2022 in Wäldi, and prior to our sampling in 2019 in Allenwiller. Natural regeneration is abundant but spatially heterogeneous at both sites.

**Fig. 1:**
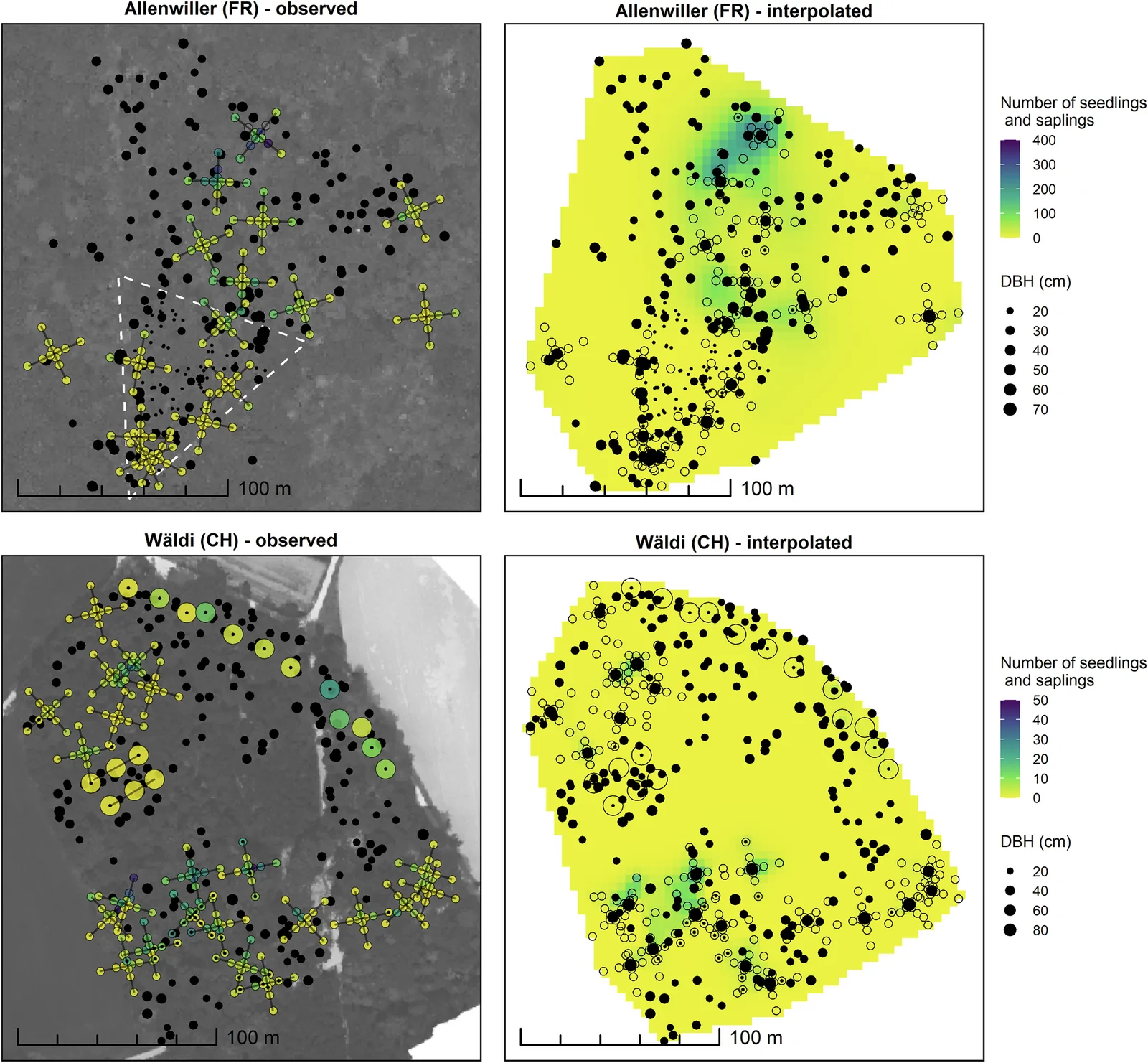
Study plots and sampling design for European beech (*Fagus sylvatica*) and Caucasian beech (*Fagus hohenackeriana*) in Allenwiller and Wäldi. Left panels show the observed number of seedlings and saplings recorded in the sampling plots, while right panels show spatially interpolated values obtained by kriging. The background aerial imagery indicates canopy structure, with lighter gray corresponding to canopy gaps or open habitats (e.g., agricultural land) and darker gray to closed forest canopy. Black dots indicate adult individuals, whereas smaller dots represent juveniles. Circles denote sampling plots: 2-m-radius plots were used in both sites; in Wäldi, additional 5-m-radius plots were included along three linear transects. In Allenwiller, the dotted polygon delineates the area within which all juvenile trees were sampled.

Between 2018 and 2019, we mapped and sampled nearly all adult trees (individuals with diameter at breast height (DBH) ≥ 19 cm) in Allenwiller (*N* = 216) and Wäldi (*N* = 226). For each site, we collected leaves or buds for genotyping and recorded GPS coordinates using a Trimble GeoExplorer GeoXH 6000. Offspring were sampled along cross-shaped transects centered on the largest adult trees following the design of Kurz et al. (2023) (Fig. 1), which enabled comparable estimates of fecundity among similarly sized trees of both species. Circular plots with a radius of 2 m were placed at distances of 2, 6, and 14 m from focal trees. Within each plot, all offspring were counted, and 20% were sampled for genotyping (reduced to 5% in plots with exceptionally dense regeneration). Offspring were classified as seedlings (<100 cm height), saplings (100–300 cm height), or juveniles (>300 cm, DBH <19 cm).

In Wäldi, offspring were sampled around the 23 largest trees (DBH > 57 cm). Additional sampling was conducted in two areas with abundant adult trees with a DBH of 15 to 19 cm using linear transects with plots of 5 m radius every 10 m. In Allenwiller, cross-transects were established around the 15 largest trees (DBH > 41 cm). In the triangular area containing clustered Caucasian adults, we sampled all juveniles, as saplings and seedlings were rare. This approach aimed to capture the main regeneration areas rather than the entire stand area (Fig. 1). To estimate regeneration density and spatial heterogeneity across the stands, we employed ordinary kriging using the GeoModels R package (Bevilacqua 2025) (Supplementary Material).

### Genotyping

We used 16 microsatellite markers originally developed for European beech and shown to be polymorphic in Oriental beech (Lefèvre et al. 2012): Csolfagus_29, Concat14, Csolfagus_05, Csolfagus_06, Csolfagus_19, Csolfagus_31, sfc_0036, sfc_1143, DE576, DUKCT, DZ447, EEU75, EJV8T, EMILY, ERHBI and FS1_15. Allele binning was conducted with TANDEM (Matschiner and Salzburger 2009) and refined manually for locus DZ447, which contained alleles differing by 1 bp. We visualized allele size distributions and rounded allele sizes to the nearest integer below the observed size. The origin of the planted Caucasian beech was verified by comparison with natural populations following Kurz et al. (2023). The number of genotyped trees per age class is shown in Table 1.

**Table 1:** Sample size of each life stage and genotype class, as assigned by NewHybrids, in the two study stands.

	Allenwiller				Wäldi			
Genotype class	Adults	Juveniles	Saplings	Seedlings	Adults	Juveniles	Saplings	Seedlings
*N*	216	96	3	540	226	70	169	184
*Pure sylvatica*	148 (68.5%)	12 (12.5%)	2 (66.7%)	398 (73.7%)	210 (92.9%)	67 (95.7%)	152 (89.9%)	115 (62.5%)
*Pure hohenackeriana*	68 (31.5%)	83 (86.5%)	–	42 (7.8%)	5 (2.2%)	–	2 (1.2%)	4 (2.2%)
*F1*	–	–	1 (33.3%)	56 (10.4%)	2 (0.9%)	3 (4.3%)	15 (8.9%)	63 (34.2%)
*F2*	–	–	–	18 (3.3%)	–	–	–	–
*BC sylvatica*	–	–	–	4 (0.7%)	–	–	–	–
*BC hohenackeriana*	–	–	–	–	–	–	–	–
*Unassigned*	–	1 (1.0%)	–	22 (4.1%)	9 (4%)	–	–	2 (1.1%)

### Hybrid genotype proportions

We used the Bayesian clustering software NewHybrids (Anderson and Thompson 2002) to estimate the posterior probability of each individual belonging to one of six genotype classes: pure European beech, pure Caucasian beech, F1, F2, and backcrosses to each parental species. To evaluate marker informativeness, we calculated the discriminatory power of each locus based on allele frequency differences between species (Fig. S1) and generated three genotype panels: all 16 loci, the eight most diagnostic loci, and the eight least diagnostic loci. We then conducted power analyses using the hybriddetective workflow (Wringe et al. 2017a). Simulated multi-generational hybrids were generated using the 30 adults with the largest DBH as the training dataset, based on STRUCTURE assignments from (Kurz et al. 2023). For Wäldi, only existing Caucasian adults were available and therefore included. Three replicate simulations were run, each with three independent Markov Chain Monte Carlo (MCMC) chains, using the *freqbasedsim_AlleleSample* function. After verifying convergence (function *nh_preCheckR*), we assessed NewHybrids’ performance across posterior probability thresholds (Fig. S2), measuring efficiency, accuracy, and power following Vähä and Primmer (2006).

We then applied NewHybrids to the full dataset using the R package *parallelnewhybrid* (Wringe et al. 2017b). Three replicate MCMC chains were run for 500,000 sweeps after a 10,000-iteration burn-in. Based on power analysis results, individuals with posterior probability ≥0.8 were assigned to a genotype class; those below this threshold were excluded from further analyses (23 individuals in Allenwiller, 11 individuals in Wäldi). All analyses were performed in R version 4.4.0 (R Core Team, 2024).

### Spring leaf phenology

To assess potential differences in flowering time between the two species, we recorded spring leaf phenology, which serves as a proxy for flowering phenology in beech (Nielsen and Muckadell 1954). Observations were made in Wäldi from 2021 to 2025 at approximately 10-day intervals during April and May, and once in Allenwiller in spring 2024, where phenology was recorded during a single field campaign. We monitored approximately 20 European beech and six Caucasian beech trees previously genotyped. Leaf development was scored on a five-stage scale from dormant bud (0) to unfolded leaf (4) following Vitasse et al. (2010).

**Fig. 2:**
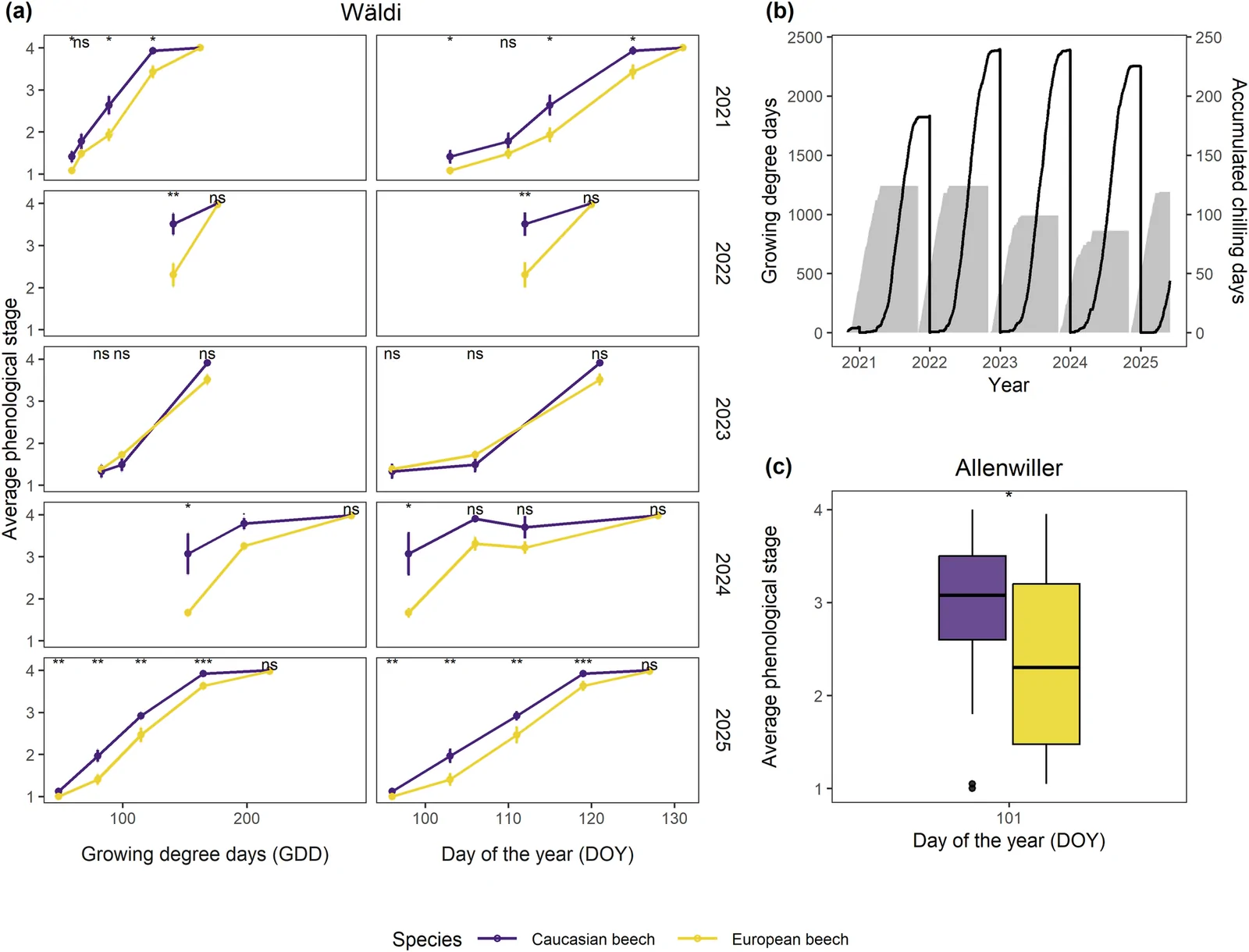
Spring leaf phenology in Wäldi (2021–2025) and Allenwiller (2024). **a** Mean and SE of spring phenological stages of European and Caucasian beech in Wäldi as a function of growing degree days (GDD) and day of year (DOY). **b** Interannual variation in GDD (black lines) and accumulated chilling days (gray areas) in Wäldi during the observation period. **c** Average phenological stage of European and Caucasian beech in Allenwiller at DOY 101 in 2024. Significance of the Wilcoxon test: **p < 0.01, *p < 0.05, n.s. not significant.

Each tree was assessed by at least two observers using binoculars (10 × 30 magnification) to record the mean canopy stage from a distance of approximately 5 m. Leaf development was analyzed relative to growing degree days (GDD) and chilling days (CD). Due to interannual climatic variation (higher GDD and lower CD in 2024–2025; Fig. 2b), we analyzed each year separately. Species differences were tested using the Wilcoxon rank-sum test on mean stage per date. For 2021 and 2023 in Wäldi, we also estimated the day of year (DOY) for stages 2 (bud burst) and 3 (leaf out) using linear interpolation to obtain comparable phenological reference dates between species. This was not possible in 2022 and 2024 because Caucasian trees were at advanced phenological stages at the first observation.

### Reproductive success and selection gradients

We investigated individual reproductive success using the neighborhood model (Burczyk et al. 2006) implemented in NM*π* (Chybicki, 2018), which integrates spatial, genotypic and phenotypic data to estimate parameters such as mean seed and pollen dispersal distances (*d*_*s*_ and *d*_*p*_), dispersal kernel shapes (*b*_*s*_ and *b*_*p*_), rates of seed and pollen immigration (*m*_*s*_ and *m*_*p*_), selfing rates (*s*_*p*_), selection gradients (*γ* and *β*). In this framework, parentage and genealogical relationships among offspring and candidate parents are reconstructed probabilistically as part of a single-step likelihood analysis. In the absence of uniparentally inherited markers, the model evaluates both possible parental configurations for genetically compatible adults and jointly infers seed and pollen contributions based on genotypic compatibility, the spatial configuration of candidate parents and the estimated seed and pollen dispersal kernels, while accounting for uncertainty in parentage assignment. Because the model assumes a single species, identical dispersal parameters were applied to both beech species.

Phenotypic proxies of reproductive success were incorporated into the parentage reconstruction. After a sensitivity analysis to optimize initial values (Supplementary Material), we tested three standardized traits: tree size (DBH), competition coefficient (Hegyi index) (Hegyi 1974) calculated within a 20-m radius using DBH of focal and neighboring trees as the size variable, and species identity, expressed as the standardized posterior probability of being European beech from NewHybrids (*P*_*syl*_). Using *P*_*syl*_ as a phenotypic trait allowed estimation of each species’ relative contribution as seed and pollen donor while accounting for abundance and spatial distribution. Significance of selection gradients was assessed as Z-scores.

Additionally, we used the absolute difference in *P*_*syl*_ between two mating trees, which measures the taxonomic distance between mating individuals. This metric captures disassortative mating (Supplementary Material). Final model estimates for both stands were obtained using maximum likelihood, the exponential power kernel(Austerlitz et al. 2004), the locus-specific genotyping error rates and the optimized initial parameters. To verify genealogy assignments, we excluded genealogies with probability < 0.8 and combined the NewHybrids classification of each individual with NM*π* output, which confirmed that all F1 offspring were assigned to parents of different species and that all parents of pure offspring were classified as pure. In contrast, F2 and backcross individuals were mostly unassigned or were assigned with probabilities below the threshold, and when assigned they were linked to pure parents, likely reflecting the absence of F1 adults among the sampled reproductive trees.

### Simulating hybridization dynamics

To explore how space, demography and hybrid fitness influence hybridization dynamics, we conducted individual-based forward-time simulations with Nemo-age v0.32.6 (Cotto et al. 2020). The simulations were designed to generate expected hybrid class distributions under neutral demographic conditions, providing a neutral baseline against which to evaluate potential selection. Deviations from these neutral expectations would indicate the action of processes such as heterosis or outbreeding depression. Parameters expected to have negligible effects on hybridization were fixed to reduce model complexity, while focusing on key parameters: pollen dispersal distance (*d*_*p*_), age at reproduction (*A*_*r*_), and hybrid fitness reduction (*W*).

To evaluate the role of the spatial configuration of trees, we compared non-spatial simulations with spatially explicit scenarios implemented via a custom simulation wrapper (https://github.com/castef/Fagus_hybrid_SSR). Space was modeled as a grid of 4 × 4 m patches (92 × 107 patches in Allenwiller and 71 × 76 patches in Wäldi), approximating the spatial scale of our circular sampling plots. Individuals occupied patch centers, with initial spacing of approximately 10–12 m, based on historical management records (Klein, 1981). Patch-level carrying capacities were calibrated to observed densities (800 in Allenwiller, 50 in Wäldi, see Supplementary Material, Fig. S4); excess seedlings and saplings were randomly removed annually. Patches representing non-beech habitats (e.g., spruce clusters or agricultural land) had zero capacity. Seedling and juvenile survival was regulated using Beverton-Holt density dependence (Beverton and Holt 2012). Seed and pollen dispersal followed two-dimensional exponential power kernels, discretized into custom *n*_*p*_ × *n*_*p*_ dispersal matrices (where *n*_*p*_ = number of patches). Non-spatial scenarios assumed uniform dispersal (equal probability 1/*n*_*p*_ per patch). We simulated three pollen dispersal distances (*d*_*p*_) – 109 m (empirically estimated), 50 m, and 20 m–while limiting seed dispersal to 50 m and pollen dispersal to 200 m.

Nemo-age enables modeling of complex forest tree demography (Dauphin et al. 2021) by allowing overlapping generations and four life stages: seedlings (year 1), saplings (years 2–8), juveniles (years 9 to reproductive maturity), and adults (reproductive maturity onward, assuming no maximum lifespan). Stage-specific survival probabilities were parametrized using values from the COMPADRE Plant Matrix Database (2023, Version 6.23.5.0; https://compadre-db.org/) (Table 2). Based on available demographic data for European, Japanese (*Fagus crenata* Blume)(Wagner et al., 2010), and Caspian beech (*Fagus caspica* Denk and Grimm) (Mirbadin and Namiranian, 2005), reproductive age (*A*_*r*_) was set to 50 years or 30 years to represent unmanaged versus intensively managed forest scenarios (Genet et al. 2010). Because harvesting processes are not implemented explicitly in Nemo-age, to approximate the effects of thinning we applied a constant adult mortality rate of 3% per year, corresponding to the average annual removal expected under the observed management regime.

**Table 2:** Parameters used in the Nemo-age simulations.

Parameter	Allenwiller	Wäldi
*Demographic Parameters*		
*N*_*p*_	9844	5396
*K*	200, 500, **800**	**50**, 200, 500
*b*	0.02, 0.01, **0.005**	0.02, **0.01**, 0.005
*s*_*p*_	0.0073	0.0032
*S*_0_	0.3	0.3
*S*_1_	0.5, **0.7**, 0.9	0.5, **0.7**, 0.9
*S*_2_	1	1
*S*_3_	0.97	0.97
*f*	1000	1000
*d*_*s*_ (m)	28.95	28.95
*Causal Parameters*		
*d*_*p*_ (m)	20, 50, 109.41	20, 50, 109.41
*A*_*r*_	30, 50	30, 50
*w*	0.1, 0.3, 0.5, 0.8	0.1, 0.3, 0.5, 0.8

Demographic parameters were identified in a preliminary optimization step (see Supplementary Material), while causal parameters were evaluated in the final simulation runs. Bold values indicate those used in the final model. *N*_*p*_ number of patches, *K* patch carrying capacity, *b* competition coefficient, *s*_*p*_ selfing rate, *S*_0_ − _3_ survival rates of offspring, seedlings, juveniles, and adults, *f* adult fecundity, *d*_*s*_, *d*_*p*_ seed and pollen dispersal distances *A*_*r*_ adult reproductive age, *w* fitness value of each DMI locus.

Species identity was simulated using 10 additive diallelic quantitative trait loci, providing sufficient resolution to track ancestry across simulated generations, with allele effects coded as −0.05 (European) or 0.05 (Caucasian), yielding phenotypic values from −1 (pure European) to +1 (pure Caucasian). F1s were heterozygous at all loci, advanced hybrids exhibited mixed allele sets. Mutation and linkage were not modeled.

To simulate hybrid incompatibilities, we used the Dobzhansky-Muller Incompatibility (DMI) model (Orr & Turelli, 2001). DMI is available in Nemo-age, and it reduces viability in individuals carrying incompatible allele combinations. Fitness (*W*) was computed as $W={(1-w)}^{{n}_{diff}}$, where *w* is the selection coefficient per incompatible pair and *n*_diff_ is the number of incompatible pairs. Four independent incompatible locus pairs were modeled with *w* = 0.1, 0.3, 0.5 and 0.8, corresponding to F1 fitness of 0.65, 0.24, 0.06 and 0.0016, respectively, with partial recovery in F2 and backcross individuals.

For each parameter combination (spatial vs. non-spatial, *A*_*r*_, *d*_*p*_, and *w*), 30 replicates were run for 200 years. All simulation scripts and ‘.ini‘ files are available at https://github.com/castef/Fagus_hybrid_SSR.

### Hybrid Index

We quantified individual introgression levels through a Hybrid Index (HI), ranging from 0 (pure parental genotype) to 1 (F1), with intermediate values (0 < HI < 1) representing advanced-generation hybrids. For observed individuals, HI was derived from NewHybrids genotype classifications; for simulated individuals, HI was computed as the proportion of heterozygous loci across the additive trait loci.

We calculated the proportion of each HI class within each sampling plot (empirical data) or patch (simulated data) values averaged across replicates. Hybrid proportion was defined as the combined proportion of F1 (HI = 1) and advanced-generation hybrids (0 < HI < 1). Patterns were evaluated at two time points: year 2020 (to match the empirical sampling) and year 2120. We assessed spatial autocorrelation in the F1 hybrid distribution using Moran’s I values implemented in the ape R package, averaging across replicates within each scenario.

## Results

### Abundance of F1 seedlings but limited persistence across later life stages

**Fig. 3:**
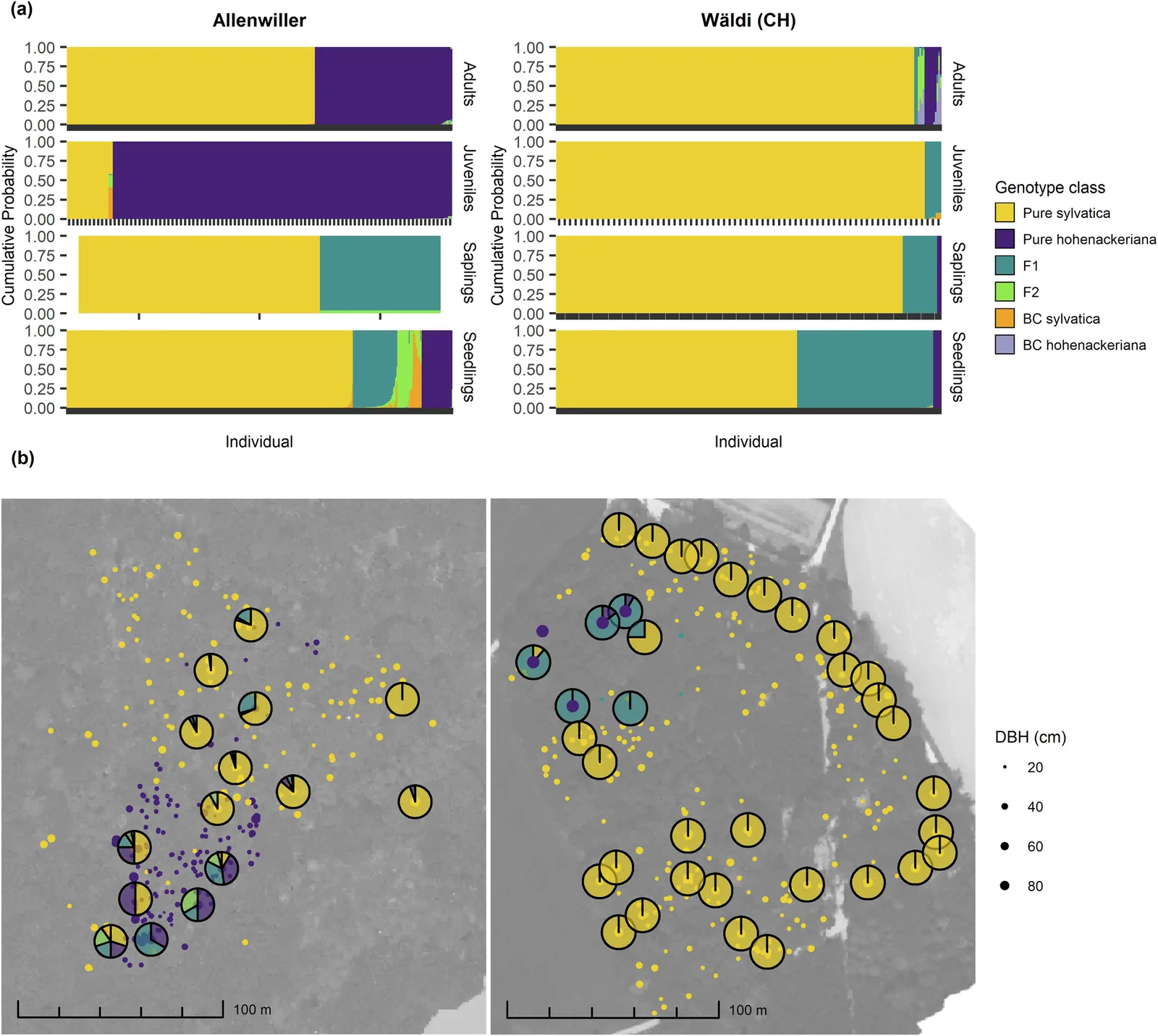
NewHybrids genotype classification of European beech (*Fagus sylvatica*), Caucasian beech (*Fagus hohenackeriana*) and hybrid individuals in Allenwiller and Wäldi. **a** Posterior probability of assignment of each individual to the six genotype classes inferred by NewHybrids. **b** Spatial distribution of genotype classes within each study plot. Colored dots represent adults (larger symbols, size proportional to the DBH) and juveniles (smaller symbols). Seedlings and saplings are displayed as pie charts summarizing the local composition of genotype classes. Genotype class legend applies to both (**a**, **b**).

NewHybrids assigned most individuals to a genotype class with high confidence (posterior probability ≥ 0.8): 100% of adults in Allenwiller and 96% in Wäldi; 98.8% and 100% of juveniles; and 96% and 99.5% of seedlings and saplings, respectively (Fig. 3a). The proportion of hybrids (F1, F2 and backcrosses combined) among all offspring classes was 12.8% in Allenwiller and 19.2% in Wäldi (Table 1). Hybridization was concentrated in seedlings, with 10.4% and 34.2% identified as F1 in Allenwiller and Wäldi, respectively. In contrast, adults were almost entirely pure, with only two F1 individuals (0.9%) detected in Wäldi and none in Allenwiller.

The spatial distribution of adult trees reflected the original planting patterns (Fig. 3b). In Wäldi, all five Caucasian beech adults were clustered in the northwest corner, surrounded by European beech, while the two F1 adults and unassigned individuals occurred along the species interface. In Allenwiller, 60 Caucasian adults formed a dense aggregation, with eight additional individuals scattered among European beech to the north and northeast.

Among juveniles, Allenwiller contained 79 pure Caucasian individuals beneath Caucasian adults and four pure European individuals, but no hybrids or admixed genotypes. In Wäldi, nearly all juveniles were European beech, except for three F1s located below Caucasian adults. The younger age classes in Wäldi followed the same spatial trend: hybrids and Caucasian offspring occurred exclusively near Caucasian adults, while the rest of the stand contained only European beech offspring. In Allenwiller, plots under Caucasian adults contained a genetic mixture: pure Caucasian beech, F1s, F2s (3.3%), backcrosses to European beech (0.7%), and a few unassigned individuals. Plots elsewhere were dominated by European seedlings. No backcrosses to Caucasian beech were found in either stand.

The 16 microsatellite loci provided high (>0.75) assignment efficiency and accuracy for pure *sylvatica*, pure *hohenackeriana*, and F1s for probability thresholds up to 0.9 (Fig. S2). Accuracy declined for F2 and backcrosses, with efficiency values dropping to 0.5 at higher thresholds. The lowest power was observed for backcrosses to *hohenackeriana* in Wäldi, likely due to the small number of reference individuals. Selecting the most informative loci markedly improved accuracy, efficiency and power for pure species and early-generation hybrids (Fig. S2), while adding more loci (regardless of informativeness) enhanced detection of backcrosses.

### Most matings are intra-specific, with fecundity driven by size and competition

**Fig. 4:**
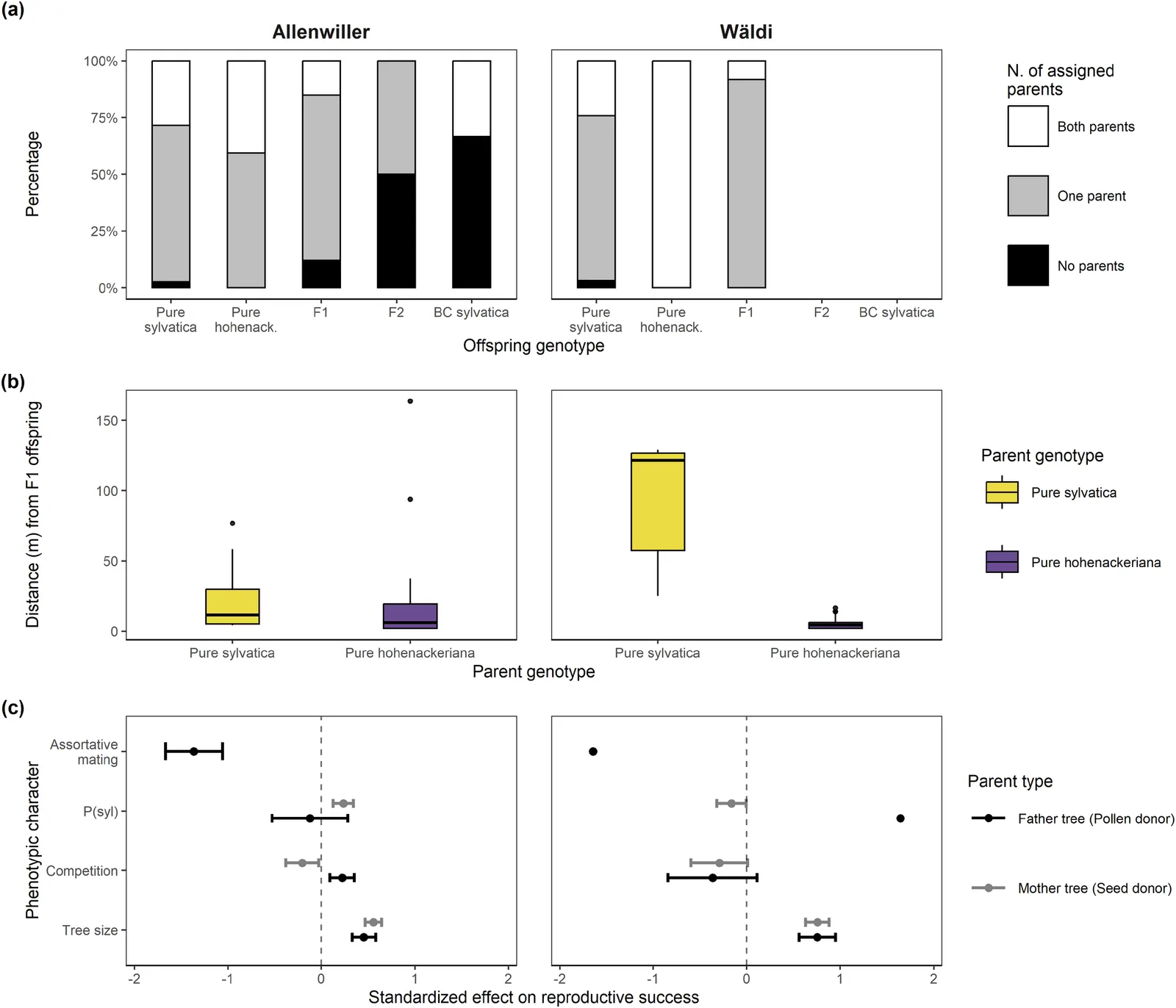
Parentage assignment, spatial patterns and drivers of reproductive success. **a** Percentage of offspring (seedlings, saplings, and juveniles) for which both, one, or no parents were assigned, grouped by genotype. **b** Distance of F1 offspring from their parents, grouped by parental genotype. **c** Standardized effects of phenotypic traits (mean ± 95% confidence intervals) on the reproductive success of seed and pollen donors, including the results of the assortative mating test from the spatially explicit mating model in Allenwiller and Wäldi (confidence intervals for the effects of *P*_*syl*_ and assortative mating in Wäldi are omitted due to space constraints).

Parentage analyses identified both parents for 26% and 21.2% of offspring in Allenwiller and Wäldi, respectively, and one parent for 66% and 76.5% (Table S3, Fig. 4a). In Wäldi, for all F1 offspring the Caucasian beech parent could be identified (Fig. S3). In Allenwiller, the model failed to assign a Caucasian parent for 40% of F1 offspring (Fig. 4a), consistent with historical felling of mature Caucasian trees. Six seedlings classified as F2 by NewHybrids had only one parent assigned within the study plot (Fig. S3), and in all cases, the identified parent was pure *F. sylvatica* or *F. hohenackeriana*. As F2 individuals require hybrid parents, this indicates that in absence of F1 adults from the sampled adult pool, the model assigned the parentage to the most likely available candidate within the plot. Parental spatial distances to F1 offspring differed between sites: in Allenwiller, both species were equally distant, whereas in Wäldi, Caucasian parents were closer to hybrid offspring (Fig. 4b).

Mating parameters were broadly similar between stands (Table 3). Seed immigration was low (*m*_*s*_ = 9% in Allenwiller; *m*_*s*_ = 4% in Wäldi), while pollen immigration was high (Allenwiller: *m*_*p*_ = 57%; Wäldi: *m*_*p*_ = 69%), consistent with wind-pollination. Selfing was negligible (*s*_*p*_ < 0.007). The pollen dispersal distance could only be estimated in Wäldi (*d*_*p*_ = 109.4 m), as the model did not converge in Allenwiller. Mean seed dispersal was short and well supported in Wäldi (*d*_*s*_ = 28 m), but less precise in Allenwiller. Fat-tailed dispersal kernels were inferred for both pollen and seeds, particularly for seed dispersal in Wäldi (*b*_*s*_ = 0.54; *b*_*p*_ = 0.85).

**Table 3:** Mating dispersal parameters and associated Z-scores for Allenwiller and Wäldi from the final NM*π* runs.

	Allenwiller			Wäldi		
Parameter	Estimate	SE	Z-score	Estimate	SE	Z-score
*m*_*s*_	0.09	0.01		0.04	0.01	
*s*_*p*_	0.0073	0.00		0.0032	0.00	
*m*_*p*_	0.57	0.03		0.69	0.03	
*d*_*s*_ (m)	119.90	44.42		28.95	3.46	
*d*_*p*_ (m)	0.00	n.c.		109.41	49.20	
*b*_*s*_	0.26	0.05		0.54	0.07	
*b*_*p*_	0.06	n.c.		0.85	0.43	
*γ*_DBH_	0.56	0.05	12.40	0.76	0.06	11.73
*β*_DBH_	0.46	0.06	7.04	0.76	0.10	7.60
*γ*_*C*_	-0.20	0.09	-2.26	-0.29	0.16	-1.88
*β*_*C*_	0.22	0.07	3.36	-0.36	0.24	-1.50
*γ*_Psyl_	0.24	0.05	4.29	-0.16	0.08	-2.01
*β*_Psyl_	-0.12	0.21	-0.59	1.64	4.13	0.40

Parameters include selfing rate (*s*_*p*_), seed and pollen migration rates (*m*_*s*_, *m*_*p*_), mean dispersal distances (*d*_*s*_, *d*_*p*_), shape parameters of the exponential-power kernel for seeds (*b*_*s*_) and pollen (*b*_*p*_), and female and male selection gradients (*γ*, *β*). “n.c.” indicates non-convergence.

Tree diameter strongly influenced reproductive success for both seed and pollen donors in both stands, with a steeper positive selection gradient for seed donors (Fig. 4c). This effect was stronger in Wäldi (*γ*_DBH_ = 0.76, Z = 11.73 for seed donors; *β*_DBH_ = 0.76, Z = 7.60 for pollen donors) than in Allenwiller (*γ*_DBH_ = 0.56, Z = 12.4 for seed donors; *β*_DBH_ = 0.46, Z = 7.04 for pollen donors). Competition significantly affected reproduction only in Allenwiller, where stronger competition reduced seed production (*γ*_C_ = −0.20, Z = −2.26) but increased pollen output (*β*_C_ = 0.22, Z = 3.36). In Wäldi, competition had a weak, nonsignificant negative effect on seed production.

Species identity also influenced fecundity, but in opposite directions across sites. In Allenwiller, European beech had greater seed donor reproductive success (*γ*_Psyl_ = 0.24, Z = 4.29), whereas in Wäldi, Caucasian beech contributed more as a seed parent (*γ*_Psyl_ = −0.16, Z = −2.01). Evidence for assortative mating was detected in Allenwiller (*β*_Psyl_ = −1.36, Z = −8.77), though not significant in Wäldi (*β*_Psyl_ = −1.64, Z = −0.40), both sites indicating preferential mating between conspecifics (Fig. 4c).

### Caucasian Beech flushes earlier than European Beech despite interannual variation

Caucasian beech consistently exhibited earlier bud break than European beech, although the magnitude of this difference varied from year to year. In Wäldi, significant differences were observed for most phenological stages in 2021 and 2025, and for early stages in 2022 and 2024 (Fig. 2a). In 2021, Caucasian beech reached bud burst (stage 2) five days before European beech (day of year, DOY: 112 vs. 117) and leaf-out (stage 3) two days earlier (DOY: 119 vs. 121). In 2023, timing was nearly identical (DOY: 109 for both at bud burst, 115 vs. 116 at leaf-out) (Fig. 2a, Table S1). Year-to-year variation in chilling and growing degree days likely contributed to these temporal shifts (Fig. 2b). Differences in bud break were more pronounced in years with more chilling days, suggesting stronger species divergence under colder winters. In Allenwiller, phenological observations in spring 2024 showed a similar pattern: on day 101, Caucasian beech trees averaged stage 2.9, compared to stage 2.35 for European beech (W = 704.5, *p* = 0.020; Fig. 2c), confirming earlier flushing in Caucasian beech.

### Neutral simulations produce site-specific hybridization patterns

**Fig. 5:**
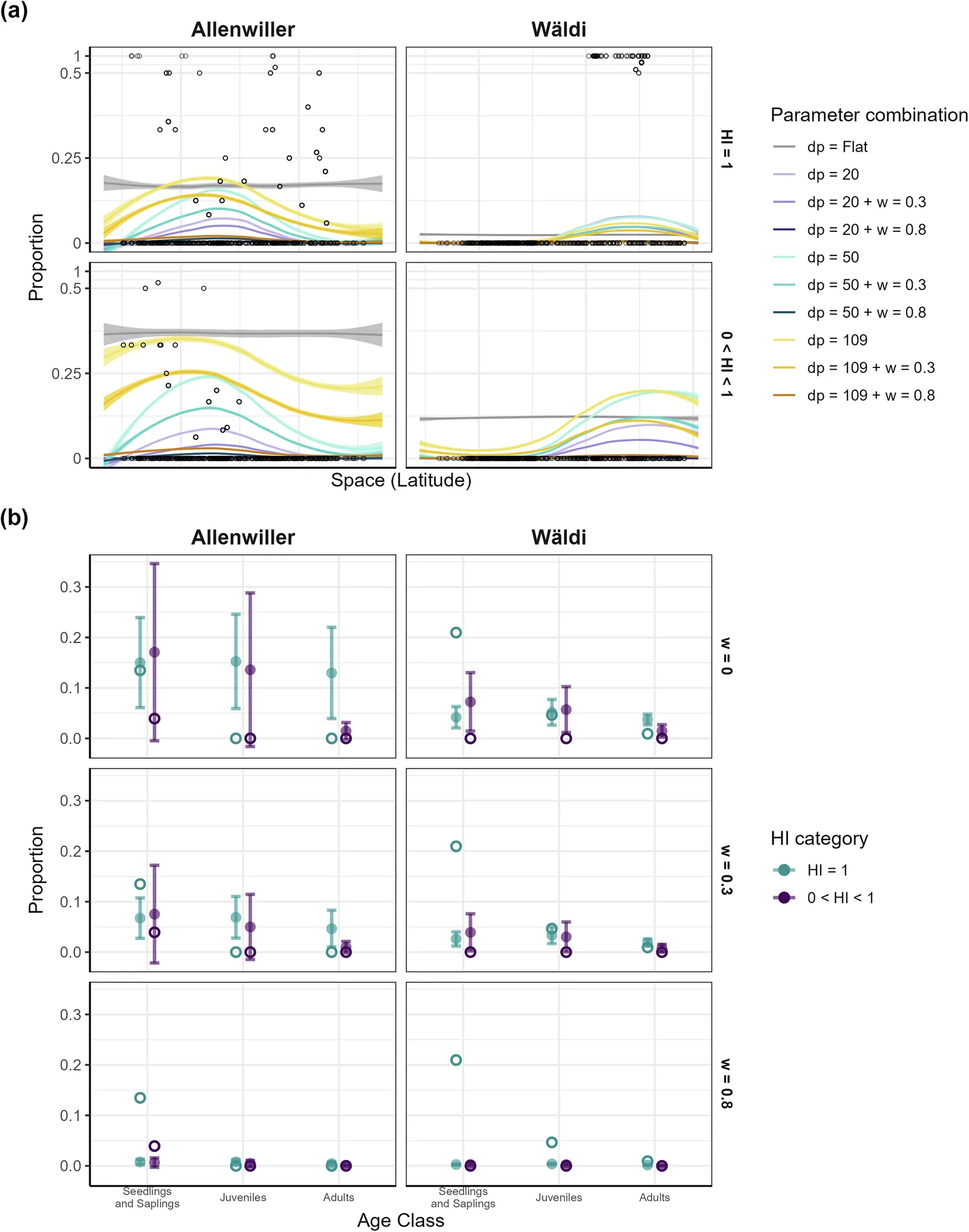
Summary of Nemo-age simulations with reproductive age *A*_*r*_ = 30 and fitness reduction *w* = 0, 0.3 or 0.8 after 100 years of simulation. **a** Proportion of F1s (HI = 1) and advanced-generation hybrids (0 < HI < 1) along the latitudinal gradient for each simulated parameter set, stand, and age class. Each point represents the mean hybrid proportion per spatial patch, averaged across simulation replicates. Observed values are shown in black: adult proportions are averaged per patch to match the model structure, while juvenile and seedling values correspond to the mean proportion within each circle plot. **b** Simulated vs. observed proportions of hybrid index (HI) categories across age classes without and with fitness reduction *w* = 0, 0.3 or 0.8 in both stands. Results for all parameter combinations are provided in the Supplementary Material.

Spatially explicit simulations showed that hybridization dynamics were strongly influenced by stand composition and spatial structure. Under neutral conditions, hybridization was widespread in Allenwiller but remained rare and spatially restricted in Wäldi (Fig. 5a), reflecting the much higher initial proportion of Caucasian beech in Allenwiller (35.6% vs. 2.53%; Fig. S6). Across all spatially explicit scenarios, including the widest dispersal kernel (*d*_*p*_ = 109), hybrids were spatially clustered near Caucasian adults, consistent with the localized distribution of the introduced species. In contrast, as expected, the non-spatial simulations produced homogeneous hybrid distributions across the landscape (Fig. 5a).

Reproductive age strongly influenced hybridization through its effect on generation time. Earlier reproduction (*A*_*r*_ = 30) produced more advanced-generation hybrids, whereas delayed reproduction (*A*_*r*_ = 50) reduced their frequency (Figs. S5 and S6). Despite these differences, the overall spatial pattern of hybridization remained similar across parameter combinations, with hybrid individuals concentrated near Caucasian adults and limited spread into the surrounding European beech stands. Across all spatially explicit simulations, hybrid frequency decreased from juveniles to adults (Fig. 5b).

### Observed hybrid frequencies deviate from neutral simulation expectations

Simulations consistently underestimated the proportion of F1 seedlings relative to the observations (Fig. 5b). This discrepancy was strongest in Wäldi (average predicted F1 = 0.02 vs. observed 0.21) and more moderate in Allenwiller (average predicted F1 = 0.06 vs. observed 0.12). In contrast, simulations slightly overestimated the frequencies of juvenile F1 and advanced-generation hybrids, typically by less than 10%.

Introducing reduced hybrid fitness improved agreement between simulated and observed genotype frequencies (Fig. 5b). At moderate selection levels (*w* = 0.3, 0.5), simulated hybrid proportions in Allenwiller aligned closely with the observed frequencies in juvenile and adult cohorts. Stronger selection (*w* = 0.8) produced overall hybrid frequencies most similar to those observed across life stages. However, even under these scenarios, simulated F1 seedlings remained less frequent than observed in the field, and no parameter combination reproduced the observed frequencies of all genotype classes simultaneously (Fig. 5b and Fig. S6a).

Reduced hybrid fitness also strongly affected spatial structure: Moran’s I declined sharply with increasing selection (from 0.1 to 0.011 in Allenwiller and from 0.21 to 0.007 in Wäldi), indicating weaker spatial clustering as hybrid survival decreased. By comparison, pollen dispersal distance had a weaker and more variable influence on spatial structure (Fig. S6b). Observed F1 distributions showed pronounced clustering (Allenwiller: Moran’s I = 0.139, *p* < 0.05; Wäldi: Moran’s I = 0.463, *p* < 0.05), exceeding the spatial structure generated by all simulation scenarios (Fig. S6b). This indicates that the observed hybridization patterns are more spatially structured than expected under neutral demographic processes alone.

## Discussion

The demographic and genetic consequences of hybridization are often difficult to observe in long-lived forest trees, yet Assisted Gene Flow (AGF) is increasingly promoted as a forest-management strategy (Aitken and Whitlock 2013; Chakraborty et al. 2024; Young et al. 2020). In the European-Caucasian beech species complex, our study, combining genetic data with spatially explicit simulations, reveals relatively high hybrid representation during early life stages followed by a strong decline across development. This pattern is supported by the sharp decrease in F1s across age classes, from high proportions among seedlings (10.4% and 34.2%) to their near absence among adults (Table 1). Despite more than a century of coexistence, hybrid and Caucasian beech spread remains restricted to the original planting zones, and advanced-generation hybrids are rare (Table 1, Fig. 3). Dispersal estimates, consistent with previous studies in beech (Oddou-Muratorio et al. 2010; Piotti et al. 2012; Westergren et al. 2023) indicate restricted gene flow. Although the long generation time and delayed reproductive maturity of beech may partly contribute to this pattern, the decline in hybrid frequency across early life stages and the spatial clustering of F1s suggest that limited hybrid persistence may also play a role. From an evolutionary perspective, introgression from Caucasian into European beech therefore appears spatially and temporally constrained under current conditions. From a management perspective, the limited spread of hybrids suggests that large-scale introgression into adjacent European beech stands is unlikely.

Hybridization in forest trees reflects interactions among ecological, spatial, and genetic factors (Petit and Hampe 2006). In our study stands, F1 seedlings were concentrated near Caucasian adults, indicating that hybrid formation is strongly influenced by the spatial distribution of the introduced species. Relative abundance and spatial structure modulate introgression dynamics: when one species is rare, heterospecific pollen may dominate the local pollen pool, increasing F1 frequencies even when reproductive barriers exist (Klein et al. 2017). Spatial clustering of rare individuals can buffer this effect by locally increasing conspecific pollen availability. In Wäldi, where Caucasian beech individuals were scarce yet spatially clustered, the relatively high F1 seedling proportion may reflect pollen limitation combined with spatial proximity. Similar density-dependent introgression patterns have been reported in several other forest tree species (Beatty et al. 2016; Buerkle 2009; Field et al. 2011; Janes et al. 2017; Lepais and Gerber 2011).

Phenological differences between species may also influence hybridization outcomes by altering the temporal overlap between pollen release and female receptivity (Whittet et al. 2017). In oaks and poplars, early-flowering or more pollen-abundant species often dominate the pollen pool (Lepais et al. 2009; Lexer et al. 2005). In our system, Caucasian beech generally exhibited earlier bud break and likely earlier flowering than European beech (Fig. 2) (von Wuehlisch et al. 2008), suggesting potential asymmetry in pollen contribution. However, the timing of male and female function in beech is poorly documented, with available observations relying largely on an early study indicating that female receptivity may precede pollen release (Nielsen and Muckadell 1954). Moreover, interindividual and interannual variation in leaf phenology, likely driven by climatic conditions, may also shift reproductive phases and thus modify opportunities for interspecific crosses.

Comparisons between observed and simulated genotype distributions further elucidate the processes shaping hybridization outcomes (Fig. 5). Although our spatially explicit simulations incorporated realistic dispersal, stand structure, and demographic parameters, no parameter combination reproduced all observed genotype proportions simultaneously. Neutral scenarios underestimated the proportion of F1 seedlings while overestimating the persistence of advanced-generation hybrids. Introducing reduced hybrid fitness improved correspondence with observed genotype frequencies, but still failed to fully capture the observed patterns across life stages. These results suggest that neutral demographic and dispersal processes alone are unlikely to explain the observed decline in hybrid representation, indicating that additional selective or life-history mechanisms may contribute.

While early-life stage heterosis could lead to the relatively high proportion of F1 seedlings, elevated F1 frequencies may also reflect substantial pollen immigration, which often exceeds expectations derived from dispersal kernels in wind-pollinated trees (Chybicki and Oleksa 2018; Hardy et al. 2019). However, because F1 seedlings arise on Caucasian seed donors, extensive external pollen flow would be expected to elevate F1 frequencies more broadly across the stand, rather than generate the observed spatial structure. Similar high F1 frequencies have been reported in northern Germany, where 56.10% of sampled seedlings were hybrids, although sampling there was partly phenotype-based (Budde et al. 2023). Together, spatial structure, species abundance, phenology, and potential early-life advantages likely all contribute to the relatively frequent formation of F1 hybrids in these stands.

The marked decrease in F1 individuals from seedlings to saplings, juveniles and adults suggests that hybrid genotypes may experience reduced persistence across development. The low frequency of advanced-generation hybrids could reflect several processes, including reduced hybrid viability, ecological selection, demographic differences, or life-history traits affecting reproductive success of the two species. The presence of advanced-generation hybrids in Allenwiller is somewhat puzzling, given the absence of adult F1 trees, and is more likely explained by intensive harvesting than by inference errors. While thinning was approximated in our simulations via a constant adult mortality rate, real-world harvesting occurs episodically and may generate stronger temporal fluctuations in hybrid frequencies than captured by our model. Differences in the age at reproductive maturity may also influence hybrid dynamics: if Caucasian beech or hybrids reach reproductive age later than European beech, such as for the related Caspian beech (Bijarpasi et al. 2020), fewer hybrid adults would contribute to reproduction, thereby limiting the production of F2 and backcross individuals independently of intrinsic fitness differences. The observed pattern may therefore reflect a combination of demographic, life-history, and selective processes.

Selection against hybrids in later life stages may arise through mechanisms such as local maladaptation or genetic incompatibilities, contributing to the generational decline in hybrid frequency. Similar dynamics occur in other tree hybrid zones, including *Populus alba* and *P. tremula* (Christe et al. 2016). In *Eucalyptus*, increased susceptibility to herbivores and pathogens or reduced viability may emerge at different life stages, causing declines in mean population fitness (Costa e Silva et al. 2012; Larcombe et al. 2016; Lopez et al. 2000; Pfeilsticker et al. 2022; Volker et al. 2008). Comparable shifts in hybrid performance across life stages have also been reported in other systems, such as catfish (Wang et al. 2024). Controlled germination and common-garden experiments using artificial crosses between European and Caucasian beech would provide valuable insights into these genetic mechanisms.

The risk of outbreeding depression increases with genetic divergence between the mixing populations (Frankham et al. 2011b). Although the phylogenetic relationships among Eurasian beeches remain partly unresolved, plastid and nuclear evidence indicate that *F. sylvatica* and *F. hohenackeriana* belong to distinct evolutionary lineages. Their divergence likely predates the split between *F. orientalis* and *F. sylvatica*, even though *F. hohenackeriana* retains some shared 5S-IGS variants with its western relatives (Cardoni et al. 2024; Denk et al. 2024). This deeper divergence may increase the likelihood of genetic incompatibilities, thereby elevating the risk of outbreeding depression following hybridization. However, because fitness was not measured directly, these interpretations remain tentative. Natural hybrid zones between *F. sylvatica* and *F. orientalis* in the south-eastern Balkans provides additional context. In these regions, smooth clinal patterns of allele frequencies indicate ongoing gene flow, while the persistence of distinct taxa suggests that selection against certain hybrid genotypes or reduced hybrid fitness may contribute to maintaining species boundaries (Gömöry et al. 2007; Hrivnák et al. 2024).

Together, our results indicate that hybridization outcomes are shaped by spatial structure, demographic processes, and fitness differences, leading to frequent formation but limited persistence of hybrids. Clarifying the relative roles of selection and life-history variation through experimental studies and spatially explicit models will be key to predicting long-term introgression dynamics and outcomes of AGF.

## Supplementary information

Supplementary Information

## Data Availability

Microsatellite genotype data, coordinates, phenological data, and results of genotype classification and parentage assignment are available in the Supplementary Material. R scripts for all of the analyses and Nemo-age .ini files from the simulations are available on https://github.com/castef/Fagus_hybrid_SSR.
