# Supplementary material for: Hybrid vigor and outbreeding depression after 100 years of replicate introduction of Caucasian beech to European forests
Source: Heredity (Edinb). 2026 Aug 10;135(9):647–59. doi: 10.1038/s41437-026-00860-7 (PMC13612591; doi:10.1038/s41437-026-00860-7)
Supplement: Supplementary file 1 [file 41437_2026_860_MOESM1_ESM.pdf]

Supplemental Information for:

Hybrid vigor and outbreeding depression after 100 years of replicate introduction of Caucasian beech to European forests

Table of Contents:

**Kriging of seedling density**	Page 2
**Performance of nuclear microsatellites panel**	Page 3
Figure S1	Page 3
Figure S2	Page 4
**Phenology**	Page 5
Table S1	Page 5
**Spatially explicit mating model**	Page 6-8
Table S2	Page 7
Table S3	Page 8
Figure S3	Page 8
**Spatially explicit forward simulations**	Page 9-13
Figure S4	Page 10
Figure S5	Page 11-12
Figure S6	Page 13
**References**	Page 14

1. Kriging of seedling density

We performed kriging on log-transformed seedling and saplings count data using the *GeoModels* R package to estimate spatial patterns of regeneration across the study area. Circle plot coordinates were used as spatial locations, and an exponential correlation model was selected to model spatial dependence. The parameters for the Gaussian random field (mean, sill, scale) were estimated via maximum likelihood, with a fixed nugget effect (0.2) to account for microscale variability. Model fit was evaluated using residual diagnostics and variogram analysis. Predictions were made on a 4 m × 4 m regular grid by applying ordinary kriging with the fitted model parameters. The predicted values were back-transformed from log scale to the original seedling density for visualization and further analysis (Figure 1).

2. Performance of nuclear microsatellites panel

To evaluate how the number and type of loci affect genotype classification, we divided the 16 microsatellite markers into two panels based on their informativeness (Figure S1). The "most diagnostic" panel included the eight loci with the greatest allele frequency differences between the two species (Csolfagus_29, Csolfagus_31, DE576, DUKCT, FS1_15, EJV8T, EMILY and ERHBI), while the "least diagnostic" panel consisted of loci with similar allele frequencies in European and Caucasian beech (Concat_14, Csolfagus_05, Csolfagus_06, Csolfagus_19, DZ447, EEU75, Sfc_36, Sfc_43). We applied the *hybriddetective* workflow and NewHybrids classification to three datasets: one with all 16 loci, one with only the eight most diagnostic loci, and one with only the eight least diagnostic loci. For each dataset, we assessed classification performance using the *hybridPowerComp* function, which calculates accuracy, efficiency and power (Figure S2). In the analysis using the least diagnostic panel, three replicates (in both Wäldi and Allenwiller) failed to converge and were therefore excluded from the final performance assessment.

Figure S1. Informativeness of nuclear microsatellite loci for distinguishing between European and Caucasian beech. Loci are ranked according to their allele frequency differences between species, used here as a measure of diagnostic power. Based on this ranking, markers were grouped into "most diagnostic" and "least diagnostic" panels, which were subsequently used to evaluate the effect of marker choice on hybrid classification performance.

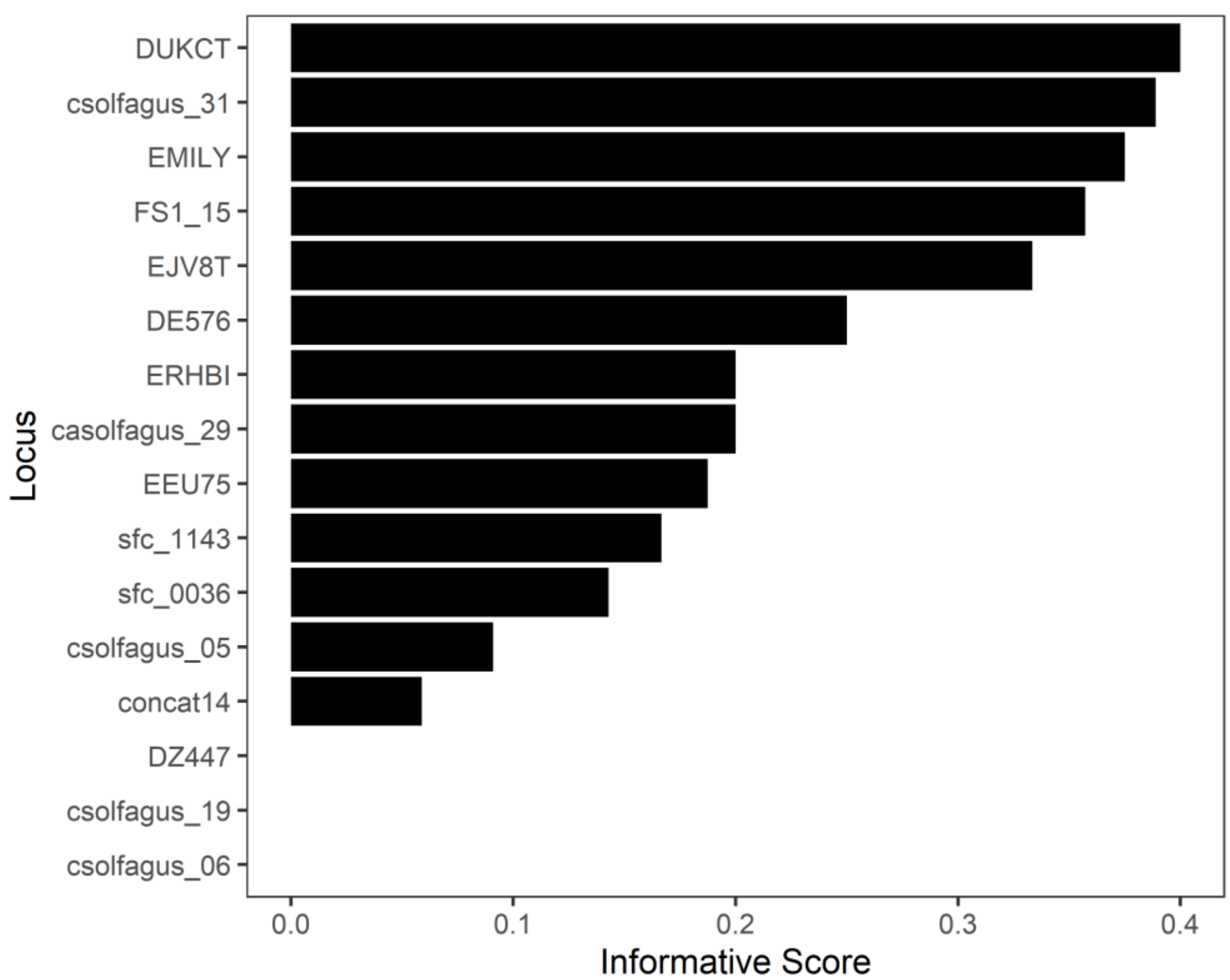

Figure S2. Accuracy, efficiency and power of the nuclear microsatellite markers for genotype assignments across posterior probability thresholds. Results are shown for three marker panels: all 16 loci, the eight most diagnostic loci, and the eight least diagnostic loci. The dotted vertical line indicates the posterior probability threshold (0.8) used for classification.

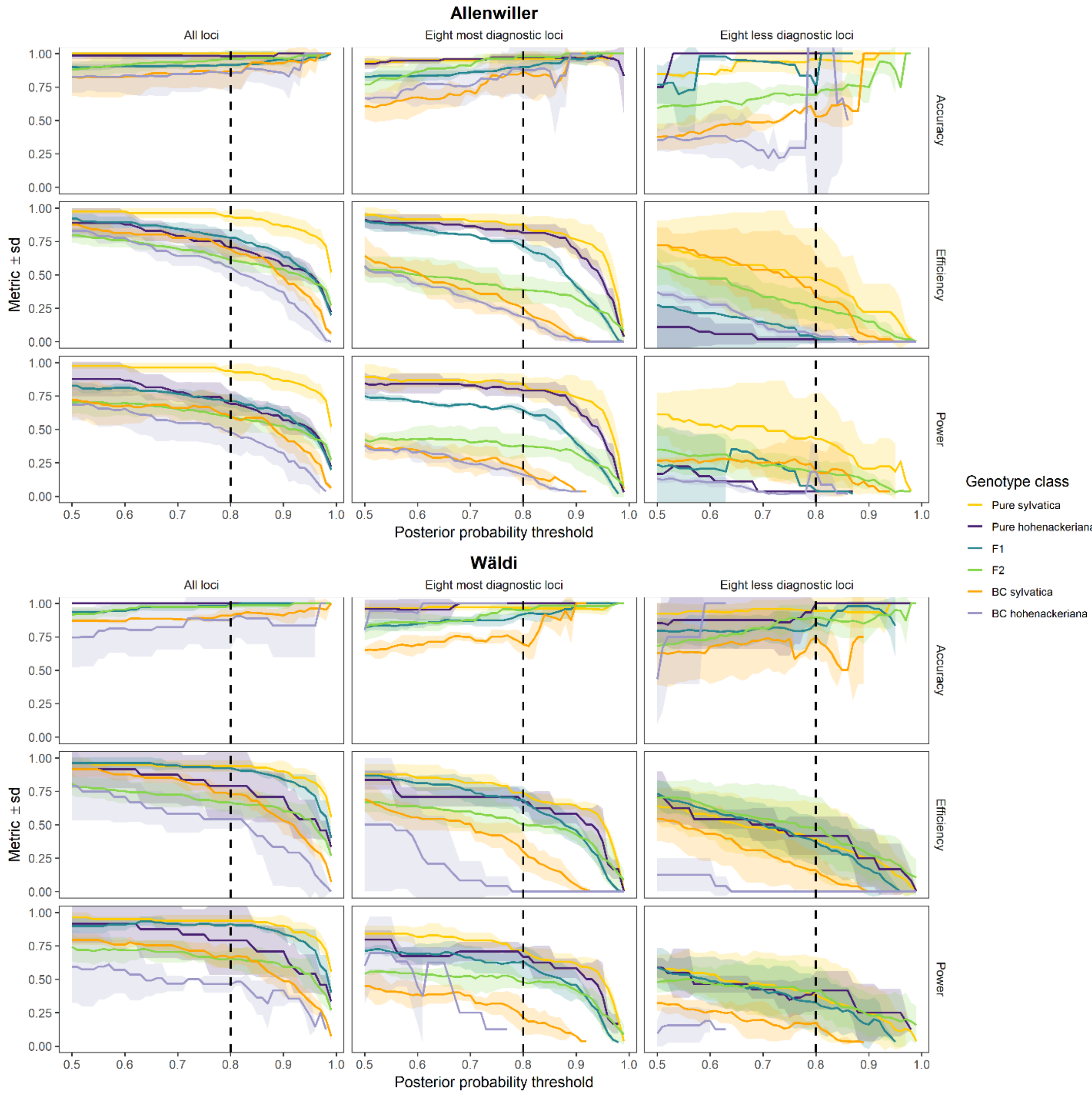

3. Phenology

Table S1. Predicted day of the year (DOY) when European and Caucasian beech reached stages 2 (bud burst) and 3 (leaf-out) in 2021 and 2023. Estimates were obtained by linear interpolation between mean phenological stage and day of year. Model fit is reported as R^2 and associated p-values.

Species	Year	Stage	Predicted DOY	R^2	P-value
European beech	2021	2	117	0.671	9.95×10^{-8}
Caucasian beech	2021	2	112	0.320	6.10×10^{-3}
European beech	2023	2	109	0.798	6.23×10^{-18}
Caucasian beech	2023	2	109	0.925	2.03×10^{-10}
European beech	2021	3	121	0.671	9.95×10^{-8}
Caucasian beech	2021	3	119	0.619	1.41×10^{-5}
European beech	2023	3	116	0.798	6.23×10^{-18}
Caucasian beech	2023	3	115	0.925	2.03×10^{-10}

4. Spatially explicit mating model

A. Parameter optimization

To optimize parameter estimation, we explored the sensitivity of the mating model implemented in NMπ by sequentially estimating each parameter while keeping all other parameters fixed, and verified the consistency of the results. We did not add any selection gradient in this testing phase.

First, we started with initial values based on previous knowledge and field observations, and calibrated the model using the ML approach. We expected considerable pollen immigration because other European beech trees surround the studied stands and because pollen can travel relatively long distances; thus, the starting value of pollen immigration (m_p) was set to 0.5. Seed immigration (m_s) was initially set to 0.03; a literature value (Westergren et al., 2023) that also corresponded to our expectation of lower seed than pollen immigration. Selfing (s_p) was set to 0.01 based on previous studies (Nielsen, 1954; Oddou et al., 2010; Westergren et al., 2023). The initial value of average pollen dispersal distance (d_p) was 1000 m, as beech is wind-pollinated and we expect pollen to travel relatively long distances (Kremer et al., 2012). The initial value of average seed dispersal distance (d_s) was 200 m, we chose a relatively high value compared to the previous studies (average d_s = 8 m in Westergren et al., 2023 and 10.5 m in Oddou et al., 2010), as the tree canopy in the stand can be up to 16 m diameter and at approximately 40 m height, thus we expect the seeds can fall to higher distances and could also be transported by animals (rodents and birds). We assumed that seed and pollen dispersal occur in all directions equally. For both seed and pollen dispersal kernels, we used the exponential power function (Austerlitz et al., 2004), as Weibull and log-normal functions gave unstable results (not shown). Additionally, the exponential power kernel is characterized by sufficient flexibility to model both "normal-like" thin tails and fat tails typically detected for wind-pollinated species (Clobert et al., 2012). The shape of the seed and pollen dispersal kernel were modeled using the shape parameters bs and bp, respectively. In the first test, these parameters were kept fixed at 0.5. We repeated the analysis using the Bayesian Hierarchical Neighborhood Model, based on the Markov-chain Monte Carlo approach, which is also implemented in NMπ; however, the algorithm failed to converge. Therefore, we performed the following steps using the ML approach.

After obtaining the first estimates, we explored the sensitivity of the mating model by changing one parameter at a time, while keeping all the other parameters fixed, and checked for the consistency of the resulting estimates. With the shape parameters fixed (i.e., they were not co-estimated), and starting values of d_s = 200 m and d_p = 1000 m, the resulting average dispersal distances were d_s = 26.35 m and 18 m, and d_p = 281.68 m and 98.72 m in Allenwiller and Wäldi, respectively. When the shape parameters were co-estimated with the other parameters, the algorithm did not converge with low pollen shape starting values (b_s = 0.01 and b_p = 0.0005, 0.001, and 0.005), which were our initial guess. The algorithm converged at higher bp starting values (0.01, 0.05, and 0.1), and the estimated bp was consistently 0.187 (Allenwiller) and 1.17 (Wäldi), while d_p was consistently approximately 12500 m (Allenwiller) and 57 m (Wäldi). This procedure was repeated for b_s, whose initial value was increased to 0.05 and 0.1. Using these initial values, the resulting estimates were consistently b_s = 0.167 and 0.43, and d_s = 250 m (Allenwiller) and 20.12 m (Wäldi). Therefore, we set both bp and bs at the starting value of 0.01 for the following runs.

We then examined the model's sensitivity to the initial mean dispersal distances. We changed the starting value of d_s from 200 m to 100 m and 10 m, obtaining the same estimates as before. We then changed the starting value of d_p to 1500 m and 500 m (with d_s = 200 m), and again, the resulting

estimates were consistent. This consistency suggested that the model is quite robust to changes in the starting values of dispersal parameters.

Per-locus genotyping errors were estimated by merging the genotype data from the two sites, thereby maximizing the data available for error rate estimation. These resulting error rates (Table S2) were used for the other runs. Since error rates were relatively high for two loci (FS1-15 = 11.9% and sfc1143 = 10.6%), we investigated their impact on parameter estimation. Using the Allenwiller dataset, we co-estimated the parameters using the same starting values as previously, but removing these two loci from the input genotype dataset (and re-estimating the genotyping errors). The resulting estimate of s_p was consistent, m_s and m_p were slightly lower (0.046 and 0.523, respectively), d_s was also lower (126.26 m), while b_s was slightly higher (0.2153). However, as these differences were minor and did not substantially affect parameter estimates, we retained both loci to avoid unnecessary loss of information.

Table S2. Per-locus genotyping error rates estimated using the NMπ software after merging genotype data from the two study sites to maximize sample size. These error estimates were subsequently used in all parentage analyses.

**Locus**	**Error (%)**
casolfagus_29	1.775
concat14	2.963
csolfagus_05	4.733
csolfagus_06	6.706
csolfagus_19	6.353
csolfagus_31	4.266
DE576	3.051
DUKCT	3.194
DZ447	4.414
EEU75	6.622
EJV8T	5.076
EMILY	5.437
ERHBI	3.101
FS1_15	11.267
sfc_0036	4.767
sfc_1143	8.787

B. Phenotypic characters and assortative mating test

Effects of the measured phenotypic characters on female and male reproductive success are assumed to be multiplicative. To ensure that individual reproductive success values are non-negative, the effects of phenotypic characters were modeled using exponential functions as:

$$\lambda_j^f = \exp(\Sigma\ \gamma_n\ z_{jn})$$

$$\lambda_{jk}^{m} = \exp[\Sigma\ \beta_n\ (c_n \times abs(z_{jn} - z_{kn}) + (1 - c_n)z_{kn})]$$

where n is the number of measured phenotypic characters, z_{kn} is the n-th phenotypic character measured for the j-th candidate parent (standardized), γ_n and β_n are slope coefficients reflecting the effect of the n-th phenotypic character on female and male reproductive success (selection gradients). C_n is a binary indicator indicating whether the n-th character's effect is incorporated as a difference between mates or as a male descriptor. When $C_n = 0$, only the male's phenotypic character value is considered. When $C_n = 1$, the absolute difference between female's and male's phenotypic character value is considered. The slope β_n becomes a measure of assortative mating given the n-th phenotypic character.
We tested the three selection gradients setting $C_n = 0$. Starting values of γ and β were set after test runs (0.5 for DBH and 0.2 for P_{syl} and competition). We also included $C_n = 1$ for P_{syl}, setting $\gamma = 0$ and $\beta = -0.2$, expecting the effect of species difference on mating to be negative.

Table S3. Summary of progeny assignment results by site obtained from the spatially explicit mating model. Pr = posterior probability of assignment to a given genealogy.

Category	Allenwiller	Wäldi
Not passed genealogies (Pr < 0.8)	324	120
Passed genealogies (Pr > 0.8)	315	302
Both parents assigned	82	64
One parent assigned	208	231
No parents assigned	25	7

Figure S3. Genotype class of progeny assigned to a single parent by the spatially explicit mating model.

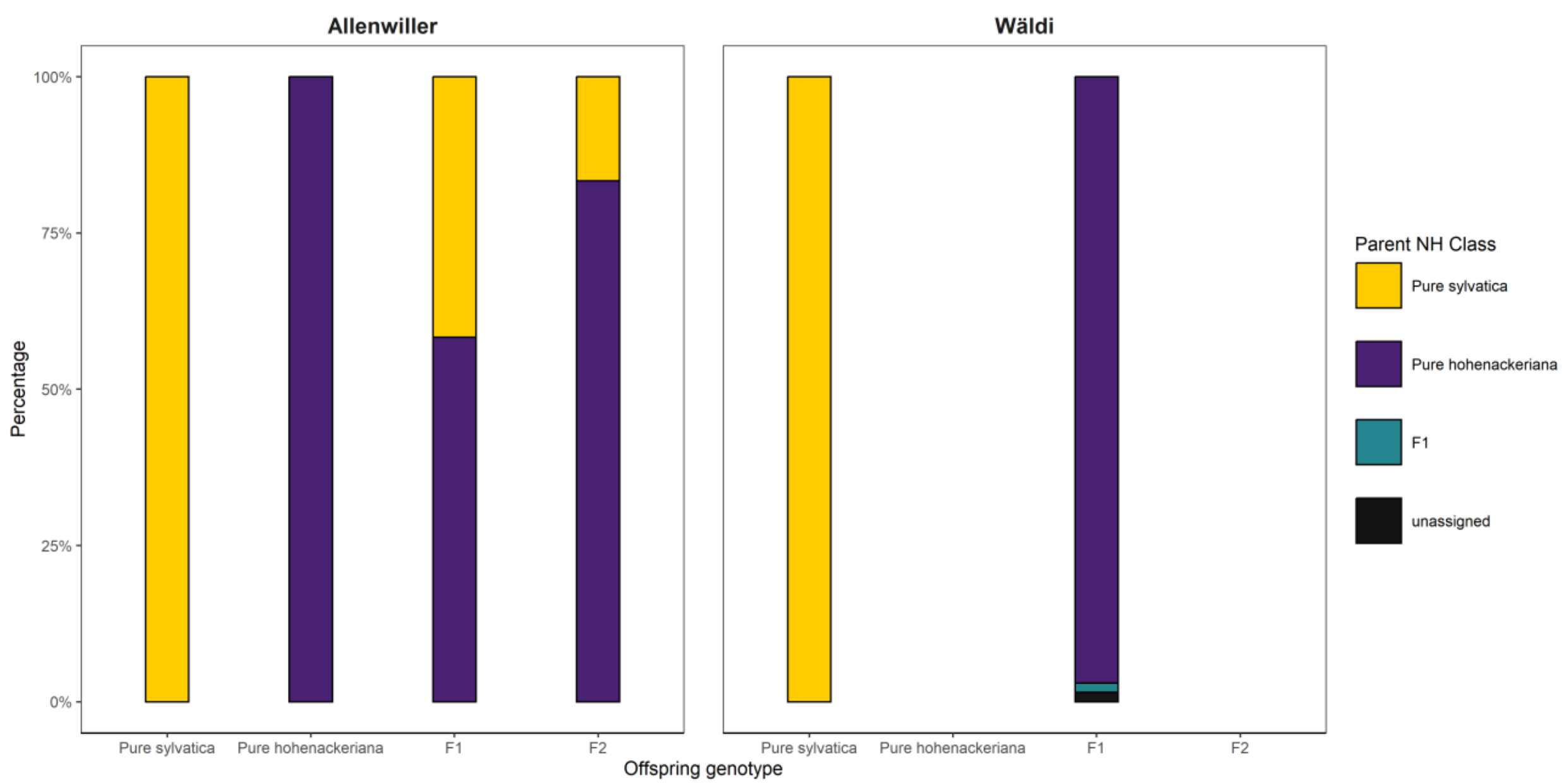

5. Spatially explicit forward simulations

A. Testing demographic parameters

To perform demographic fitting, we tested a range of demographic parameters based on empirical observations and literature data. Our aim was to obtain simulated population age structure similar to the real situation, therefore, we started from a number of adults closely matching the reality (53 Caucasian trees and 96 European trees in Allenwiller, five Caucasian trees and 192 European trees in Wäldi), as previously described. For simplicity, we did not include the few adult Caucasian beech trees observed among European beech trees in Allenwiller. Starting trees were regularly placed in the grid reproducing the clustering observed in reality, regularly distributed with approximately 10-12 m spacing. These first scenarios were based on historical records and management information of the two stands. Age structure is divided into four discrete life stages: stage 0 = seedlings (year 1), stage 1 = saplings (years 2–8), stage 2 = juveniles (years 9 to reproductive maturity), and stage 3 = adults (from reproductive maturity onward, with an infinite lifespan).

We tested different values of four demographic parameters potentially affecting population age structure: patch carrying capacity (K = {50, 200, 500} in Wäldi and K = {200, 500, 800} in Allenwiller), seedling survival (S_1 = {0.5, 0.7, 0.9}), adult reproductive age (A_r = {30, 50}), and competition coefficient acting on saplings and seedlings (b = {0.02, 0.01, 0.005}). For each parameter combination and each stand, we ran 10 replicates for 150 generations.

In preliminary runs, we also varied fecundity (f) and adult survival (S_3), but observed no differences in the output (results not shown), so we fixed those at biologically reasonable values. Estimates for other demographic parameters were based on literature (Trasobares et al., 2016; Maringer et al., 2021; Wilkens et al., 2021) and kept fixed throughout all simulations (see Table 2).

To analyse the output, we extracted the number of individuals per stage class in patches corresponding to the sampled area. We then scaled these numbers by the sampled area and monitored them over time (Figure S4). For each stand and parameter combination, we compared the scaled number of individuals at generation 70 with observed values using chi-square tests. The combination with the lowest Chi value was selected as best matching reality and used for the main simulations. A summary of tested parameters is shown in Table 2.

Figure S4. (a) Initial spatial configuration used in Nemo-age simulations, reflecting the observed species composition and clustering in each stand. (b) Temporal dynamics of demographic parameter fitting. Continuous lines represent the simulated number of individuals per life stage (scaled by the sampling area and squared root transformed for better visualization), while dotted line indicate the observed number of individuals in the real stands scaled by the relative sampling area. K = patch carrying capacity, S_1 = seedling survival, b = competition coefficient, A_r = age at first reproduction.

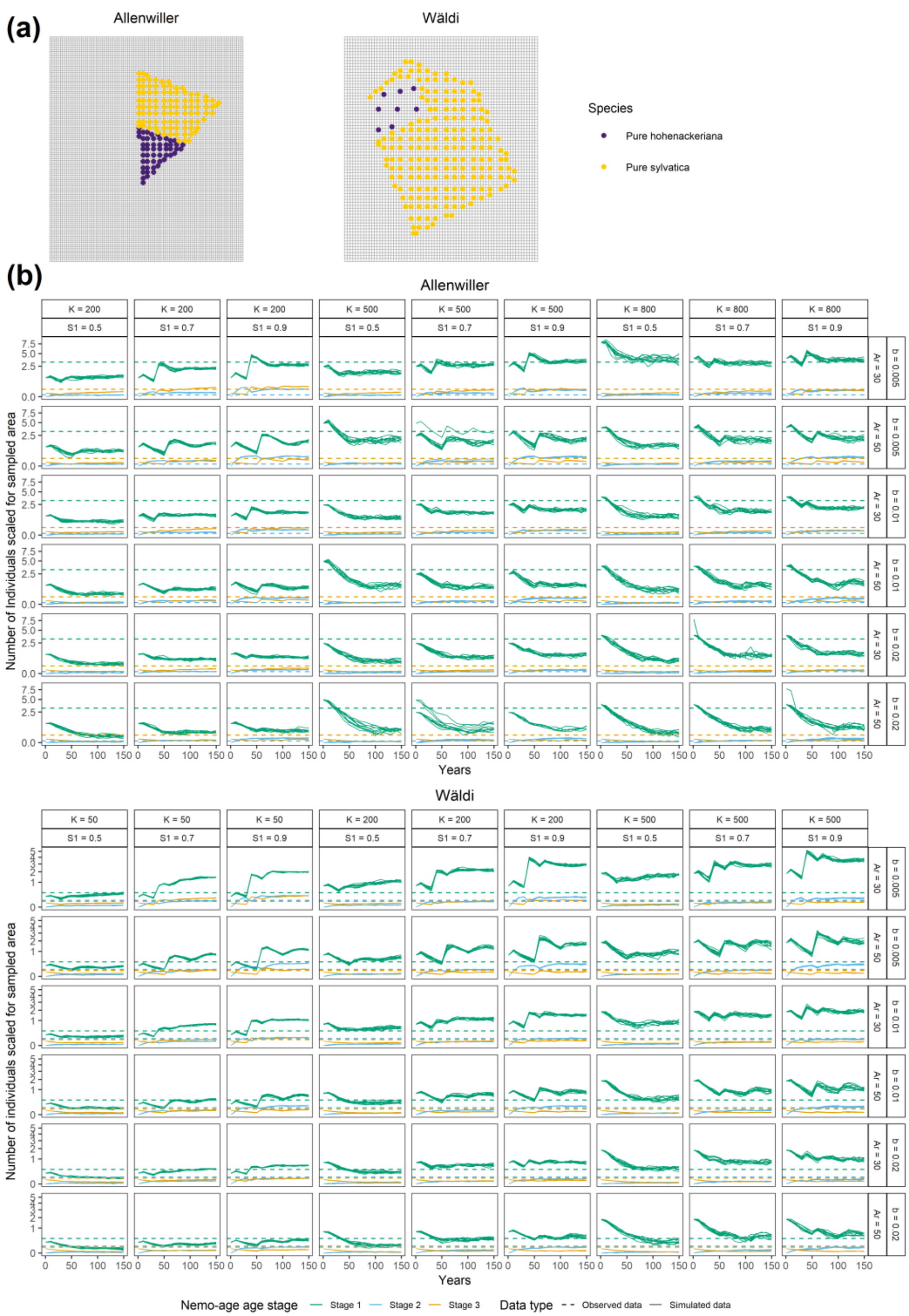

B. Simulated patterns of HI across parameter combinations

Figure S5. Simulation patterns of Hybrid Index (HI) for 2020 and 2120 in (a) Allenwiller and (b) Wäldi across all parameter combinations. Colors indicate the dominant HI category within each patch, while color intensity reflects its relative frequency (lower intensity indicates a mixture of HI categories within the patch). HI = 0 corresponds to Pure individuals, HI = 1 to F1, and 0 < HI < 1 to advanced generation hybrids (F2 and backcrosses). The proportion of hybrids for each parameter combination (averaged across replicates) is reported within each panel.

a)

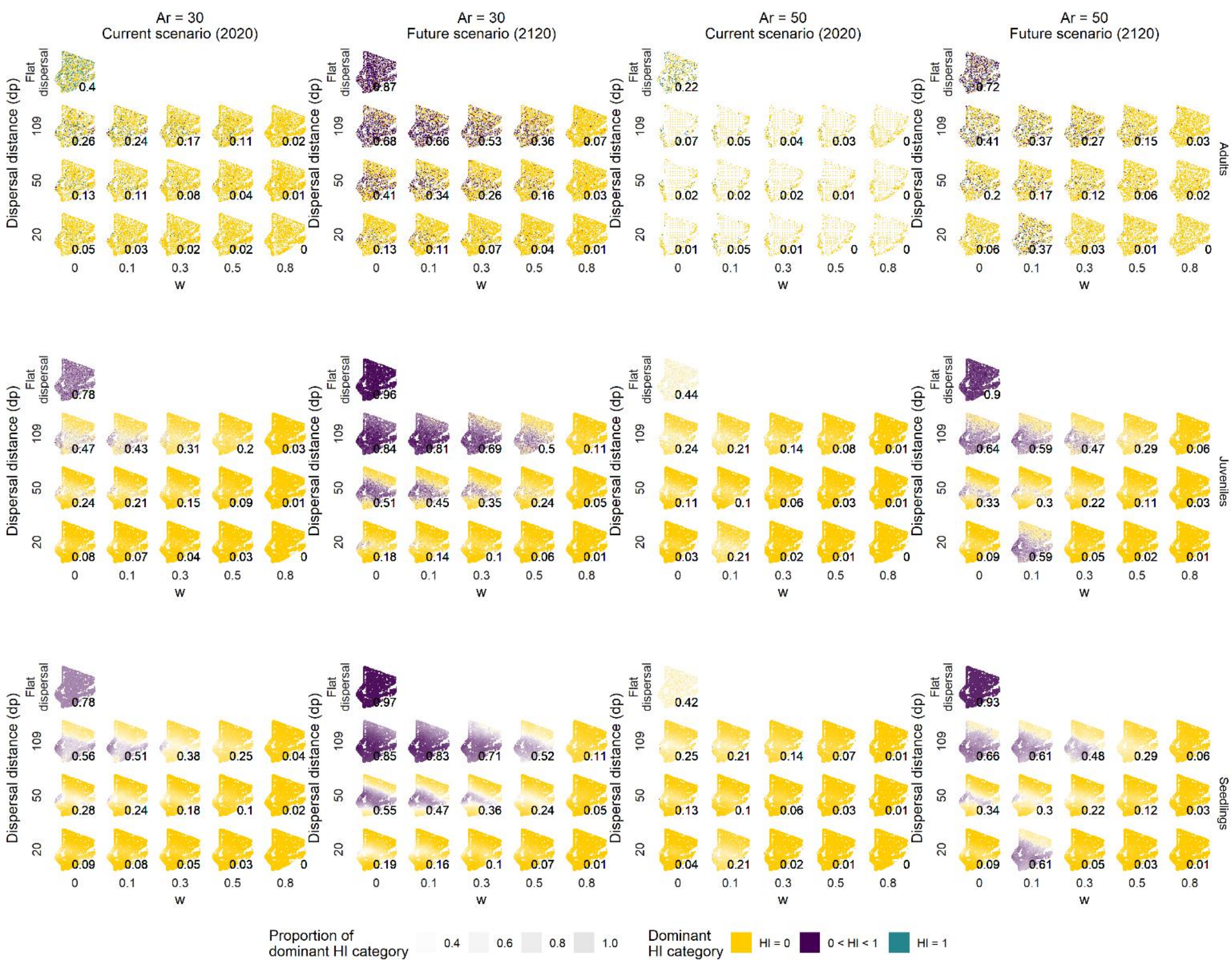

b)

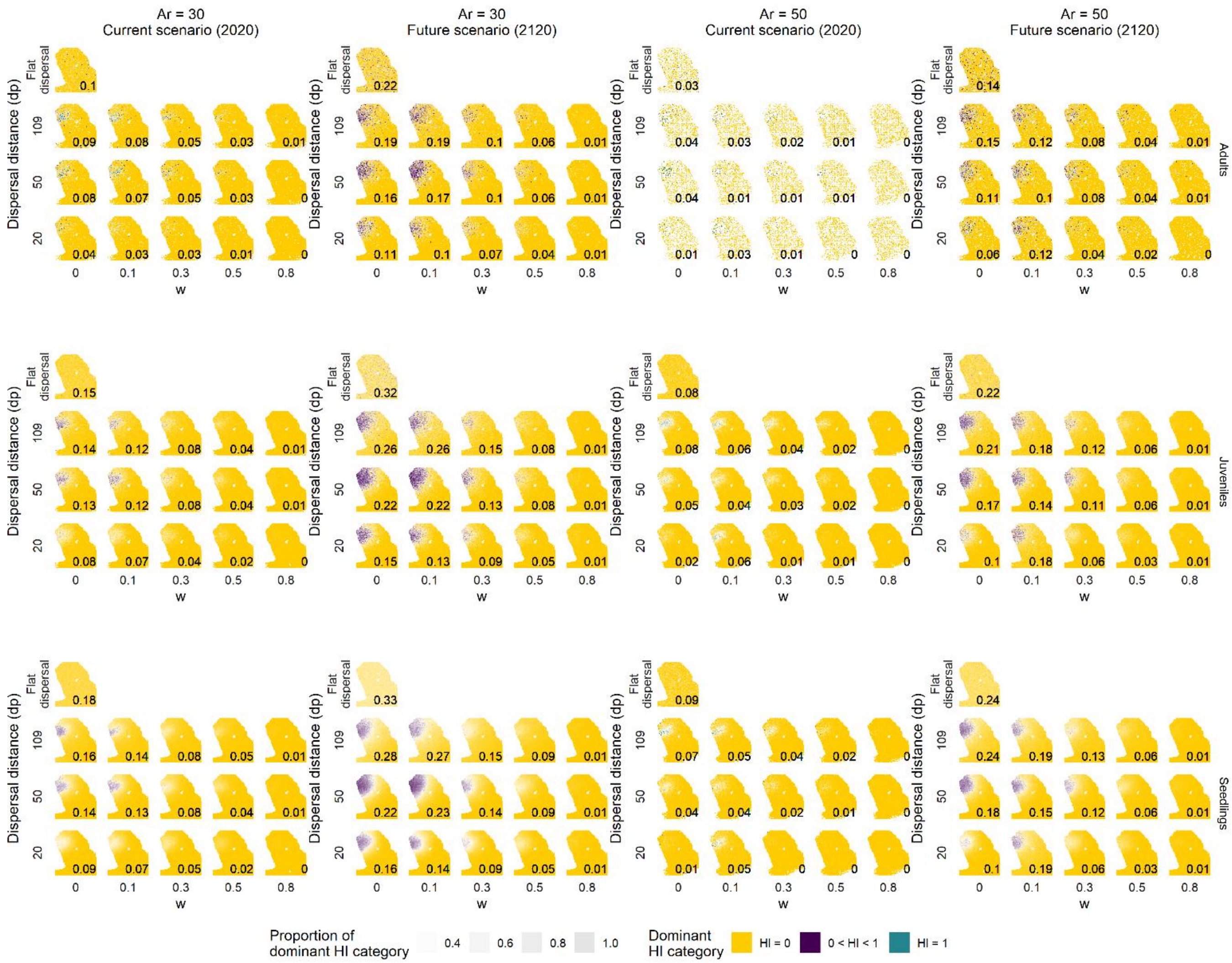

Ar = 30
Current scenario (2020)
Ar = 30
Future scenario (2120)
Ar = 50
Current scenario (2020)
Ar = 50
Future scenario (2120)
Flat dispersal
Dispersal distance (dp)
109
50
20
0
0.1
0.3
0.5
0.8
w
Adults
Juveniles
Seedlings
Proportion of dominant HI category
0.4
0.6
0.8
1.0
Dominant HI category
HI = 0
0 < HI < 1
HI = 1

Figure S6. Summary of Nemo-age simulation results. (a) Proportion of F1 hybrids (HI = 1, left) and advanced-generation hybrids (0 < HI < 1, right) along the latitudinal gradient for each parameter combination, stand, and age class. Top panel correspond to simulations with A_r = 30, while bottom panels refer to simulations with A_r = 50. Each point represents the mean hybrid proportion per spatial patch, averaged across simulation replicates. Observed values are shown as black points: adult proportions are averaged per patch to match the simulated structure, whereas for juveniles and seedlings, each point corresponds to the mean observed proportion within a circular plot.
(c) Spatial autocorrelation of the F1 distribution for different parameter combinations in Allenwiller and Wäldi, estimated using Moran's I. Colors represent pollen dispersal distances (d_p), while color intensity is reflects parameter *w*. Dotted lines indicate observed Moran's I values. Legends apply to all the panels.

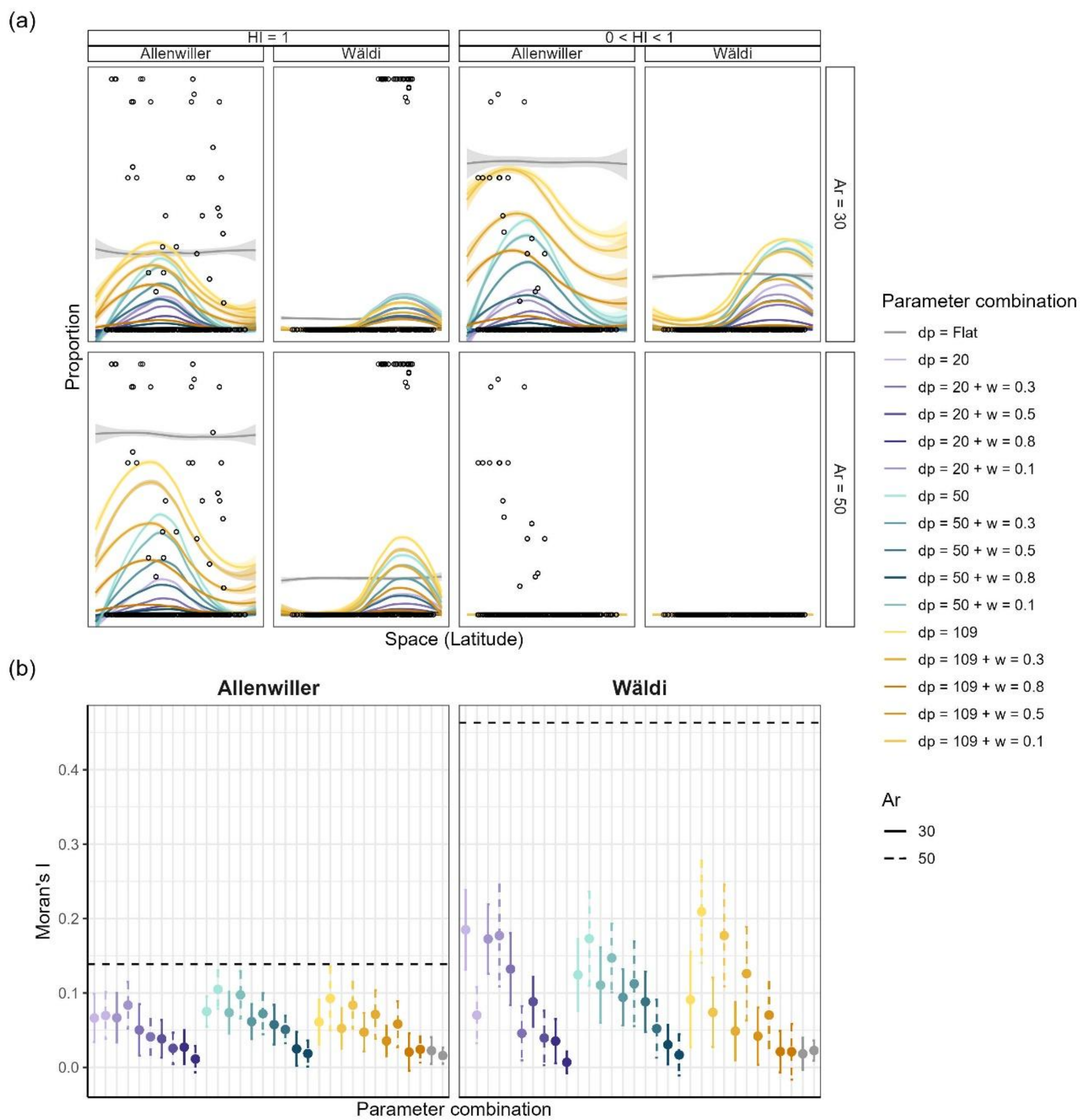

References

Austerlitz F, Dick CW, Dutech C, Klein EK, Oddou-Muratorio S, Smouse PE, and Sork VL. Using genetic markers to estimate the pollen dispersal curve. Molecular Ecology, 13(4):937–954, 2004.

Bajc M, Archambeau J, Damjanić R, Theraroz A, Kraigher H, Oddou-Muratorio S, and González-Martínez S C. Low but significant evolutionary potential for growth, phenology and reproduction traits in European beech. Molecular Ecology, 2023.

Bigler C, Trasobares A, Zingg A, and Walthert L. A climate-sensitive empirical growth and yield model for forest management planning of even-aged beech stands. European Journal of Forest Research, 135:263–282, 2016.

Clobert J. Dispersal ecology and evolution. Oxford University Press, 2012.

Kremer A, Ronce O, Robledo-Arnuncio J J, Guillaume F, Bohrer G, Nathan R, Bridle J R, Gomulkiewicz R, Klein E K, Ritland K, Kuparinen A, Gerber S, and Schueler S. Long-distance gene flow and adaptation of forest trees to rapid climate change. Ecology Letters, 15(4):378–392, 2012.

Maringer J, Stelzer A-S, Paul C, and Albrecht A T. Ninety-five years of observed disturbance-based tree mortality modeled with climate-sensitive accelerated failure time models. European Journal of Forest Research, 140(1):255–272, 2021.

Nielsen P Chr and de Muckadell M S. Flower observations and controlled pollinations in *Fagus*. Zeitschrift für Forstgenetik und Forstpflanzenzuchtung, 3:6–17, 1954.

Oddou-Muratorio S, Bontemps A, Klein E K, Chybicki I, Vendramin G G, and Suyama Y. Comparison of direct and indirect genetic methods for estimating seed and pollen dispersal in *Fagus sylvatica* and *Fagus crenata*. Forest Ecology and Management, 259(11):2151–2159, 2010.

Wade M J and Kalisz S. The additive partitioning of selection gradients. Evolution, pages 1567–1569, 1989.

Wilkens J F and Wagner S. Empirical survival model for European beech (*Fagus sylvatica* L.) seedlings in response to interactive resource gradients and (a-) biotic conditions within an experimental canopy gap study. Forest Ecology and Management, 499:119627, 2021.
